# Interspecific hybridization in tomato influences endogenous viral sRNAs and alters gene expression

**DOI:** 10.1186/s13059-022-02685-z

**Published:** 2022-05-21

**Authors:** Sara Lopez-Gomollon, Sebastian Y. Müller, David C. Baulcombe

**Affiliations:** 1 Department of Plant Sciences, University of Cambridge, Cambridge, CB2 3EA UK; 2 Present Address: Alva Genomics, Otto-Devrient-Strasse 6, 07743 Jena, Germany

**Keywords:** Small RNAs, Hybridization, Changes in gene expression, Endogenous pararetroviruses, Transposable elements, DCL2 (Dicer-like 2), RNA silencing

## Abstract

**Background:**

Hybridization is associated with the activation of transposable elements and changes in the patterns of gene expression leading to phenotypic changes. However, the underlying mechanisms are not well understood.

**Results:**

Here, we describe the changes to the gene expression in interspecific *Solanum* hybrids that are associated with small RNAs derived from endogenous (para)retroviruses (EPRV). There were prominent changes to sRNA profiles in these hybrids involving 22-nt species produced in the DCL2 biogenesis pathway, and the hybridization-induced changes to the gene expression were similar to those in a *dcl2* mutant.

**Conclusions:**

These findings indicate that hybridization leads to activation of EPRV, perturbation of small RNA profiles, and, consequently, changes in the gene expression. Such hybridization-induced variation in the gene expression could increase the natural phenotypic variation in natural evolution or in breeding for agriculture.

**Supplementary Information:**

The online version contains supplementary material available at 10.1186/s13059-022-02685-z.

## Background

The domestication of many crops is associated with mutations of genes affecting, for example, branching, seed dispersal, or fruit size that marked a major phenotypic transition [1]. Superimposed on saltatory [2–4] changes associated with these key domestication genes, there has been hybridization and then progressive changes under selection over several generations leading to phenotypes that are improvements on the progenitors. These progressive changes have been attributed to “transgressive segregation” in which recombination generates optimal allele combinations. There could be, for example, complementation of defective or missing genes from one parent by functional homologs from the other. However, there is now evidence of more complex mechanisms resulting in phenotypes that are outside the parental range. These mechanisms are associated with chromosome rearrangements [2–5], transposable element mobilization [6, 7], DNA methylation [3, 8, 9], and small (s)RNAs including micro (mi)RNA and small interfering (si)RNAs [10].

Our hypothesis that sRNAs influence gene expression in hybrids is based on findings that they are associated with transposable elements and that they can act either in *cis* or *trans* through their ability to base-pair with sequence motifs in a target gene or RNA [11]. Transposons are the most variable components of genomes in different varieties within a species or between species [12], and we predicted that sRNAs from one parental genome of a hybrid would find novel targets in the other genome. Changes to gene expression could be due to the effects at the transcriptional or posttranscriptional level depending on which of the several sRNA silencing pathways is involved with these transposable element RNAs [11].

A previous test of this hypothesis was based on introgression lines produced by recurrent backcross of *Solanum lycopersicum* × *S. pennellii* to the maternal parent [10]. These plants had sRNA and gene expression profiles outside the parental range, but the backcrossing strategy would have eliminated hybridization-induced epigenetic or genetic changes unless they were in the small retained regions of the *S. pennellii* genome [13]. Therefore, as an alternative approach, we crossed the same *Solanum* species and analyzed the selfed F4 progeny for changes to the gene expression and sRNA relative to the parents.

Our findings reported here are that gene expression and sRNA changes in independent lineages were non-random: changes to gene expression and sRNA were more similar in independent lineages than expected by chance. Among the affected sRNA loci, there was a higher proportion of those corresponding to endogenous pararetroviruses (EPRVs) than other genome features, and the associated sRNAs from these loci were 22-nt-long rather than the more abundant 21-nt and 24-nt species. These 22-nt sRNAs are produced through a minor sRNA silencing pathway, mediated by the endonuclease DCL2 (Dicer-like 2) that was previously associated with virus infection [14]. There was also a striking correspondence between changes to the gene expression in the hybrids and a *dcl2* mutant. Our findings indicate that hybridization induces changes to gene expression that persist into the F4 generation. It is likely that these changes are due to perturbation of DCL2-mediated sRNA silencing and that EPRVs are involved.

## Results

**Fig. 1:**
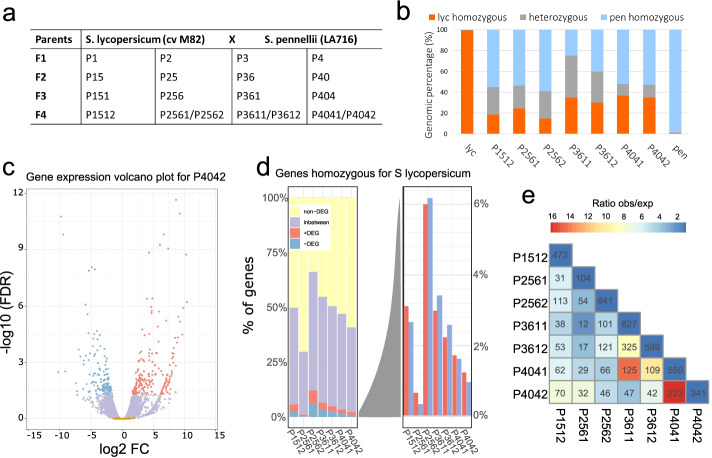
Gene expression analysis of *lyc* homozygous transcribed regions in hybrids. **a** Parental lines (*S. lycopersicum* and *S. pennellii*) and offspring (from F1 to F4), each column being a separate F1 lineage. For F2–F4, each plant keeps the number of the parental plant adding one digit. The number of digits indicates the generation (i.e., P4042 is F4 generation, progeny of plant P404). **b** Percentage of homozygous transcribed regions for *S. lycopersicum* (orange), *S. pennellii* (blue), or heterozygous transcribed (gray) in each of the F4 plants and parental lines. **c** Volcano plot showing the fold change (FC) in genes upregulated (+ DEG, red), downregulated (− DEG, blue), “in-between” (lilac), and not differentially expressed (non-DEG, yellow) in *lyc* homozygous transcribed regions for P4042. Thresholds: DEG, FDR < 0.05; “in-between” genes, 0.05 < FDR < 0.9; non-DEG, FDR > 0.9. **d** Percentage of genes in each category calculated from the total of genes in the lyc homozygous transcribed region, being each bar an individual F4 plant compared to *lyc* parent. The right-hand panel shows an expanded view of the + DEG (red) and − DEG (blue). **e** Overlap matrix showing the number of *lyc* DEG genes shared among the F4 hybrids. The color scale shows the ratio of observed over expected genes shared in a pairwise combination

The fertile F1–F4 progeny *S. lycopersicum* (M82) × *S. pennellii* (LA716) (*lyc* × *pen*) (Fig. 1a) had variable phenotypes (Additional file 1: Fig. S1) that were mostly within the parental ranges and transient in the F1, but some were transgressive (beyond the parental range) and maintained into the F4 (Additional file 1: Fig. S1b). This level of phenotypic variation is typical of hybrid populations and justified the use of these hybrids for a more detailed analysis of gene expression and sRNAs.

Our interest was in persistent and heritable changes rather than transient effects that could be linked to heterosis, and for that reason, we focussed our initial analysis on the F4 generation. We also focused on expressed genes and sRNA loci (SL) in homozygous transcribed regions (Fig. 1b, Additional file 1: Fig. S2) rather than attempt comparison of homozygous and heterozygous components of the hybrid genome. We validated our genotyping approach based on RNA-Seq data in our parental lines, obtaining a prediction of 99.6% *lyc* homozygosity for *S. lycopersicum* and 98.6% *pen* homozygosity for *S. pennellii* (see the “ Methods” section, Fig. 1b). The general trend in seven F4 plants from four different lineages was that homozygosity in the F4s is greater for *pen* (average 48%) than for *lyc* (average 28%) and that heterozygosity (average 24%) is greater than anticipated (12.5%) (Fig. 1b). The deviation from expected Mendelian inheritance may be due to the selection for fertility that was low in the F1 and F2 generations.

### Patterns of gene expression in hybrids deviated from those in the parents

From seven F4 RNA-Seq datasets [15], we identified up- or downregulated genes in homozygous transcribed regions (+ DEG and –DEG, respectively (FDR < 0.05)) (Fig. 1c, d, Additional file 1: Fig. S3a, Additional file 2: Table S1) relative to the corresponding parent. There were also genes that were not differentially expressed (non-DEG (FDR > 0.9)) and an “in-between” class that we could not assign to any of the other three classes with high confidence.

There were 6000 to 14,000 *lyc* homozygous transcribed genes in each of the F4 datasets of which 1.2 to 12.6% were differentially expressed and equally divided between those that were up- or downregulated, respectively (Fig. 1d). There were similar patterns and numbers of differentially expressed genes from the *pen* homozygous transcribed regions (Additional file 1: Fig. S3a). The sets of differentially expressed *lyc* genes were most similar between sibling F4 plants, but there was also more overlap in differential expression than expected by chance in the four independent lineages (Fig. 1e). From this observation, we conclude that hybridization-induced mechanisms affecting gene expression are non-random: a differentially expressed gene in one plant is also likely to be similarly affected in independent lineages.

### sRNA loci from endogenous viruses were affected in hybrids

**Fig. 2:**
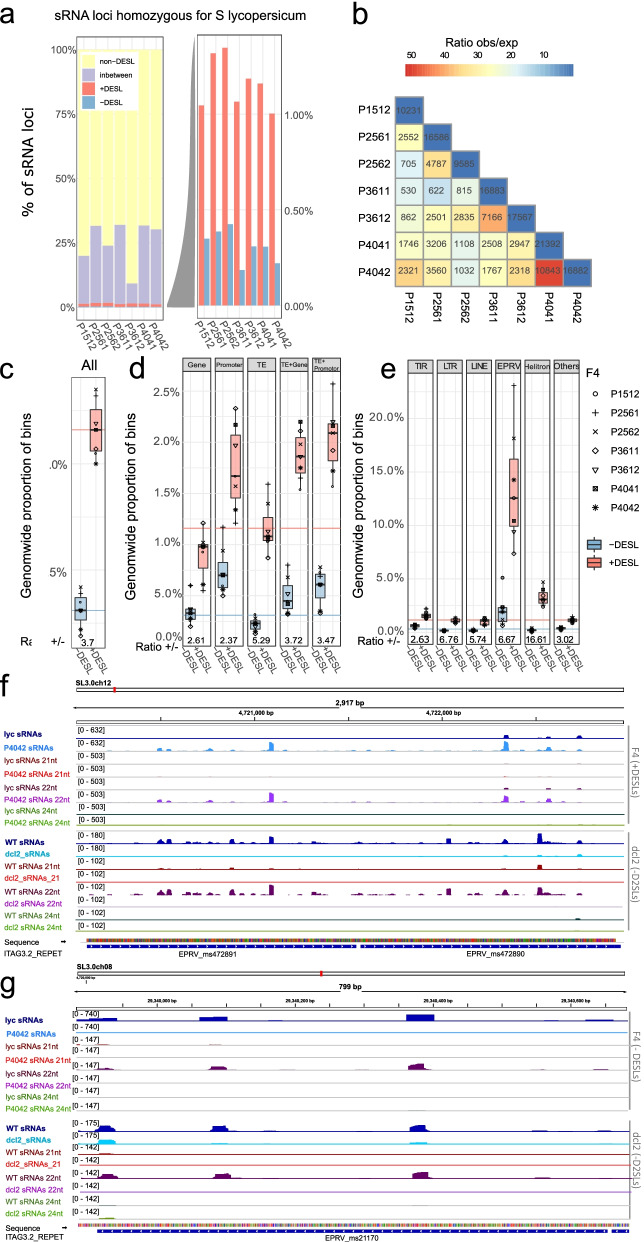
sRNA analysis of *lyc* homozygous transcribed regions in hybrids: **a** *S. lycopersicum* genome is divided into 200-nt bins (sRNA loci). Percentage of sRNA loci in each group (F4s vs *lyc* parent) calculated from the total number of sRNA loci in *lyc* homozygous transcribed regions. Groups are sRNA loci upregulated (+ DESL, red), downregulated (− DESL, blue), “in-between” (lilac), and non-differentially expressed (non-DESL, yellow). The right-hand panel shows an expanded view of the + DESL (red) and − DESL (blue). Thresholds: DESL, FDR < 0.05; “in-between,” 0.05 < FDR < 0.9; non-DESL, FDR > 0.9. **b** Overlap matrix showing the number of *lyc* DESL shared among the F4 hybrids. The color scale shows the ratio of observed vs expected sRNA loci shared in a pairwise combination. **c**–**e** Box plot showing the percentage of *lyc* sRNA loci that map **c** genome-wide, **d** to each genomic feature, **e** to TE order that are + DESL or − DESL. The ratio of + DESL/ − DESL is shown at the bottom. Each plant is represented by different symbols. Box plot elements: box limits, upper and lower quartiles; center line, median; whiskers, from each quartile to the minimum or maximum. For comparison, colored lines are the genome percentage values for + DESL (red) and − DESL (blue) (from **c**). **f**, **g** Genome browser snapshots of two genomic regions with **f** sRNA loci upregulated in the F4 and downregulated in *dcl2* (+ DESL/ − D2SL) and **g** sRNA loci downregulated in the F4 and *dcl2* (− DESL/ − D2SL). Tracks show sRNA mapping (blue) and filtered for 21 nt (red), 22 nt (purple), and 24 nt (green) for *lyc* and P4042 (top tracks) and WT and *dcl2* (bottom tracks). The numbers in brackets show the scale (normalized number of reads) for each track. The P4042 data are from this paper and *dcl2* raw data from [18]

To test the possibility that transposon-derived sRNAs could mediate altered patterns of gene expression in hybrids [10, 16], we characterized sRNA profiles [17] in the parental and hybrid lines. The sRNA loci (SL) were distributed widely, and as with the gene expression profiles, they were divided into four classes based on whether they were up- or downregulated (+ DESL and − DESL), not differentially expressed (non DESL) or “in-between” when comparing each F4 data with the corresponding parent (Fig. 2a, Additional file 1: Fig. S3b, Additional file 3: Table S2). The differentially expressed SL accounted for 1.2–1.8% of the total (FDR < 0.05) corresponding to thousands of loci. More of these loci were up- rather than downregulated. As with the differentially expressed genes, the hybridization-induced changes to SLs were most similar between sibling F4 plants, but there were also more coincident patterns of differential expression than would be expected by chance in the independent lineages (Fig. 2b): a differentially expressed SL in one plant was also likely to be similarly affected in independent lineages.

High-level genome features (genes, promoters, transposable elements) overlapped similarly with differentially expressed *lyc* SLs (*lyc* DESLs, Fig. 2c, d), and for each of them, there were more up- rather than downregulated loci following the genome-wide pattern (Fig. 2c). There is a similar profile of differentially expressed SLs in the *pen* homozygous transcribed regions although the bias to upregulation was less pronounced than in *lyc* (Additional file 1: Fig. S3b-d).

However, analysis of transposable element types revealed that endogenous pararetroviruses (EPRVs) and helitron SLs deviated from the genome-wide pattern. An exceptionally high proportion of SLs overlapping EPRVs and helitrons were differentially expressed (about 13% and 3%, respectively) (Fig. 2e, f). For EPRV elements, there was an overrepresentation of both up- and downregulated SLs, whereas for helitrons, the deviation from the genome-wide pattern was specifically with upregulated loci. In the *pen* genome, there was a similar over-representation of differentially expressed SLs among the EPRVs (Additional file 1: Fig. S3e). Genomic alignment of the sRNA sequence data implicates subsets of the EPRV elements in this differential expression, but they do not belong to a specific clade of the phylogeny tree (Additional file 1: Fig. S4).

### Differential expression of DCL2-dependent sRNA loci in hybrids

**Fig. 3:**
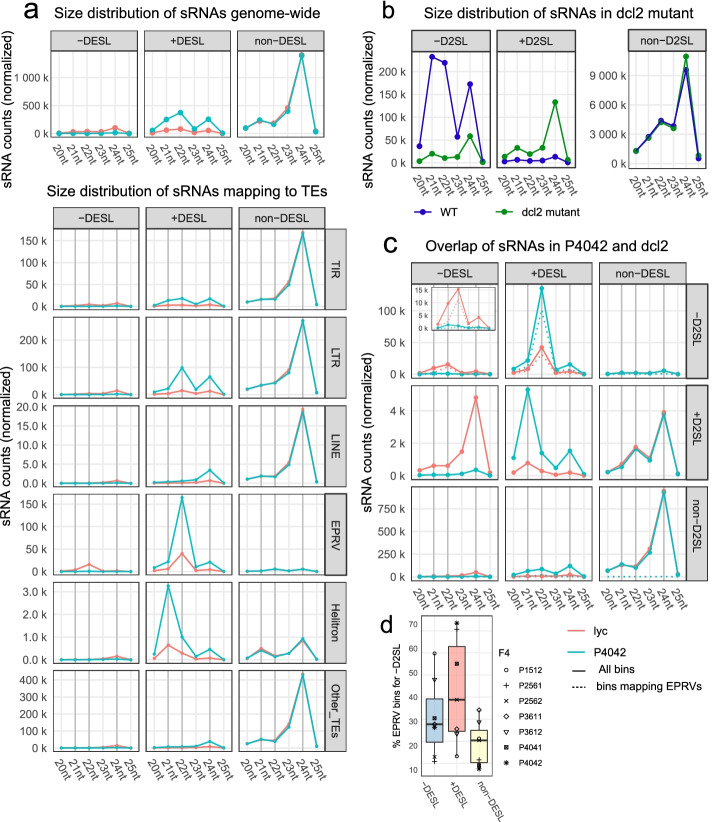
sRNA size distribution and DCL2 dependency. **a** Normalized sRNA counts for P4042 (blue line) compared to *lyc* (red line) plotted by size for − DESL, + DESL, and non-DESL (top panel) and mapping to each TE order (bottom panels). **b** Normalized sRNA counts plotted by size in *dcl2* mutant (green) compared to its WT (dark blue), from − D2SL (downregulated sRNA loci), + D2SL (upregulated sRNA loci), and non-D2SL (non-differentially expressed sRNA loci). **c** Normalized sRNA counts plotted by size in P4042 from − DESL, + DESL, and non-DESL that overlap − D2SL, + D2SL, and non-D2SL in *dcl2*. The red line, sRNA counts for *lyc*; blue line, sRNA counts for P4042; solid line, counts for all sRNA loci in each group category; dotted line, counts for sRNAs in each category that map to EPRVs. Inset with expanded *y*-axes for − DESL/ − D2SL. **d** EPRV contribution to each − D2SL group in **c** top panel in each F4 plant is represented by different symbols. Box plot elements: box limits, upper and lower quartiles; center line, median; whiskers, from each quartile to the minimum or maximum

Small RNA size classes (21, 22, or 24 nt) are associated with distinct RNA silencing pathways [19]. Most of the non-differentially expressed sRNAs were from the 24-nt class (non-DESL, Fig. 3a, Additional file 1: Fig. S5a) whereas the differentially expressed SLs especially those in the upregulated class (+ DESL, Fig. 3a, Additional file 1: Fig. S5a) included 21-nt and 22-nt species. The 22-nt sRNAs were predominant in differentially expressed SL mapping to TIR and LTR transposons and, strikingly, to EPRVs (Figs. 2f and 3a, Additional file 1: Fig. S5b).

The predominant mechanism for 22-nt sRNA production involves DCL2 [18, 20, 21], and correspondingly, the sRNA loci in WT tomato that decrease in *dcl2* (-D2SL) have abundant 21-nt and 22-nt species whereas those that increase (+ D2SL) or are not affected (non-D2SL) are predominantly 24 nt (Fig. 3b). DCL2 clearly plays a major role in the F4 plants because sRNAs mapping to the loci that increase or decrease (+ or − DESL, respectively) also map predominantly to the loci that are dependent on DCL2 (− D2SL) with the 22-nt size class being prominent in all lines (Figs. 2f, g and 3c, Additional file 1: Fig. S5c). Of these DCL2-dependent sRNAs from the SL that are up- or downregulated in the F4 plants, a high proportion map to EPRVs (Figs. 2f, g and 3d, dotted line in + and − DESL/ − D2SL panels of Fig. 3c and Additional file 1: Fig. S5c).

This pattern of DCL2 dependency (− D2SL), 22-nt abundance, and EPRV representation was specific to the sRNAs that increased or decreased in the F4 plants (from + or − DESL) (Fig. 3c, d). The sRNAs that were not affected in the hybrids (non-DESL) were also not dependent on DCL2 (non-D2SL) and were predominantly 24 nt. Those that were changed in the hybrids (+ or − DESL) and increased rather than decreased in *dcl2* (+ D2SL) were generally of lower abundance than the DCL2-dependent loci (− D2SL), and they were a mixture of 21- and 24-nt species (Fig. 3c, Additional file 1: Fig. S5c). These various patterns indicate, therefore, that the up- or downregulated sRNAs in the F4 hybrids (+ and − DESL) are produced predominantly by DCL2, and they largely correspond to EPRV loci.

To find out whether these changes to sRNA expression occurred immediately after the *lyc* × *pen* hybridization, we analyzed the differentially expressed SL from the *lyc* parent and the F1–F4 generations (Additional file 1: Fig. S6). In each instance, there were up- and downregulated SLs in the F1. For the upregulated loci, the sRNA reads gradually increase from the F1 to the F4 generation, but for the downregulated loci, there was little further decrease in the expression after the F1.

### DCL2 affects gene expression in *lyc × pen* hybrids

Associated with the sRNA changes in *dcl2*, there were also up- and downregulated genes (+ D2G and − D2G, respectively) [20, 22]. If there is a causal link involving DCL2, 22-nt sRNAs, and changes in the gene expression in the *lyc* × *pen* hybrids, we predicted an overlap of the genes that are differentially expressed in the F4s (DEG) and in *dcl2* (D2G) relative to the parental or wild-type plants. To test this prediction, we reanalyzed the published *dcl2* and WT RNA-Seq datasets [20, 22] (Additional file 2:Table S1) and compared the differentially expressed genes with the up- and downregulated genes in our F4 hybrids.

**Fig. 4:**
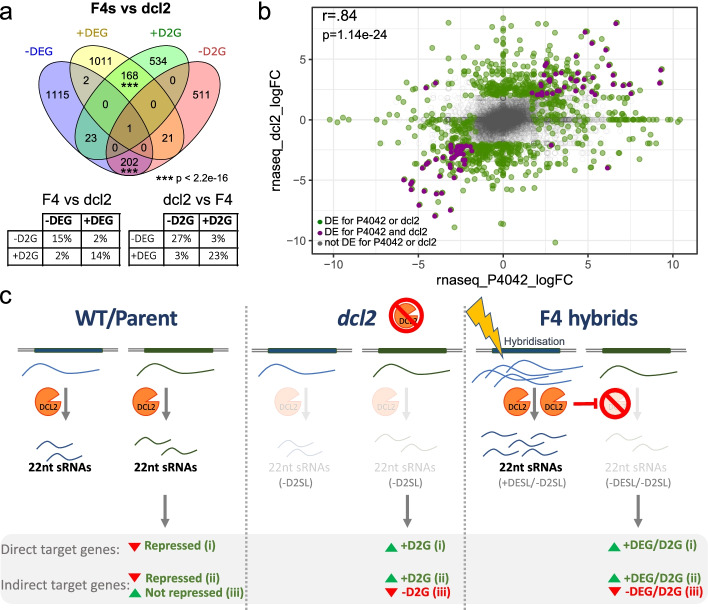
Common response in the change of gene expression in the F4 hybrids and *dcl2* mutant. **a** Venn diagram of the consolidated DEG for the F4s (compilation of + / − DEGs in at least one F4 plant) compared to differentially expressed genes in *dcl2* (+ / − D2G). Statistical association determined by Fisher’s exact test*** two-tailed *p* < 2.2e − 16. Tables showing the percentage of DEGs in the F4 (being 100%) shared with *dcl2* (left) and for DE genes in *dcl2* (being 100%) shared with F4 (right). **b** Scatter plot of the fold change (FC) in the gene expression in P4042 vs *dcl2*. Gray, non-DE; green, DE genes for P4042 or *dcl2*. Purple: DE genes for both P4042 and *dcl2*. Pearson correlation coefficient value (*r*) for DEG and D2G genes (purple genes), *p* = *p*-value. **c** The diagram shows subsets of sRNA loci (SL) that are dependent on DCL2 and + DESL or − DESL depending on whether they are over- or under-expressed in the *lyc* × *pen* hybrids. In the WT/parental plants, we envision that sRNAs from these loci cause direct sRNA silencing of target genes (i) or can also have an indirect effect if the target is an activator (ii) or repressor (iii). In the *dcl2* mutant, these direct or indirect effects would be reversed because the sRNAs would not be produced. In the *lyc* × *pen* hybrid, the wild type/parental silencing effects would also be reversed if the overexpressed + DESL sRNAs are able to compete with the − DESL sRNAs for components of the DCL2 sRNA silencing pathway. Most of these + DESL and DCL2-dependent sRNAs correspond to EPRVs (Fig. 3c)

This analysis (Fig. 4a, b, Additional file 1: Fig. S7a,b) revealed an extraordinary coincidence in the patterns of gene expression affected by *dcl2* and the *lyc* × *pen* hybridization both in terms of the gene identity and the quantitative effect on the expression level. The coincident identity was reflected in 23% of the genes upregulated in *dcl2* (+ D2G) also increasing in the F4s (+ DEG) and 27% of the downregulated genes in *dcl2* (-D2Gs) decreasing in the F4s (− DEGs). These values were highly non-random (*p*-value < 2.2e − 16): only 3% of the genes upregulated in *dcl2* (+ D2Gs), for example, overlapped with downregulated genes in the F4s (− DEGs) and 3% of the − D2Gs coincided with + DEGs. The correlated quantitative effects were reflected in the near-linear relationship in the level of up- or downregulation in the F4s and *dcl2* (Fig. 4b, Additional file 1: Fig. S7b). From these patterns, we conclude that the hybridization of *lyc* × *pen* perturbed the DCL2-mediated pathway of sRNA silencing and thereby influenced the profile of gene expression in the F4 progeny.

Among the differentially expressed genes, 10% are transcription factors, including several WRKY factors [23], key regulators of biotic and abiotic stress, or BBX/CONSTANS [24] genes that are central regulators of developmental processes such as pigment synthesis, seedling photomorphogenesis, flowering time, and hormonal pathways. This pattern suggests that EPRV and the associated sRNAs are key regulators in the transduction of a stress signal into a cellular response, leading to modified phenotypes. These genes are plausible contributors to the transgressive phenotypes in the F4 plants (Additional file 1: Fig. S1), and the data are, therefore, consistent with our starting hypothesis that sRNAs and transposons influence hybridization-induced changes in the gene expression and transgressive phenotypes. The refinement of this hypothesis, reported here, is the identification of the affected genes and the involvement of EPRV and DCL2 in the underlying mechanisms.

## Discussion

We show here that changes in the gene expression following the hybridization of *lyc* and *pen* are associated with perturbation of sRNA pathways involving DCL2. This DCL2-related mechanism affects at least 168 out 1203 + DEGs in the F4 and 202 out of 1343 − DEGs (Fig. 4a). These values are likely to be underestimated because we used stringent criteria for DE assignment in the *dcl2* and F4 lines and because 24.9% of the genome was not *lyc* homozygous transcribed for any F4 plant (Additional file 1: Fig. S2) and was excluded from the comparison with *dcl2*.

We emphasize that other mechanisms are also likely to be involved in the hybridization-induced changes to the gene expression*.* Some of these could, in principle, involve sRNAs from the various types of transposon loci that are more numerous than EPRV in the tomato genome including LTRs, helitrons, and other transposons (Figs. 2e and 3a, c, d). However, given that (Fig. 3 and Additional file 1: Fig. S5) the DCL2-dependent EPRV sRNAs account for a major part of the DESL sRNAs, is it likely that the EPRVs are a significant influence on the hybridization-induced changes to the gene expression in the hybrid lines. For that reason, we focus this discussion on the involvement of EPRVs and DCL2.

This hybrid-specific and DCL2-related effect on the gene expression is not likely to involve reduced levels of DCL2 because 22-nt sRNAs were increased rather than decreased in the F4 lines (Fig. 3c) suggesting a functional DCL2. Also not consistent with a change to DCL2, DCL2 mRNA levels do not vary to the same extent in the F4 lines (Additional file 2:Table S1); the levels of the DCL2 regulator miR6026 remain in the parental range [18] (Additional file 3: Table S2) in the F4s, and there was no consistent change to the miR6026-triggered sRNAs from the DCL2 loci in the F4 lines [18] (Additional file 3: Table S2). One hypothetical scenario is that the highly abundant 22-nt sRNAs from the + DESLs out-compete or antagonize the 22-nt DCL2-dependent sRNAs produced in the parental plants (Figs. 3c and 4c). A consequence of this competition or antagonism would be that the parental pattern of DCL2-dependent silencing is blocked and disruption to gene expression in the hybrid would be, as observed (Fig. 4a, b), similar to that in a *dcl2* mutant.

Gene expression changes in the *dcl2* mutant involve both up- and downregulation [20]. Upregulation would be a direct effect of sRNA silencing if the target RNAs in a wild-type plant bind to a DCL2-dependent sRNA (Fig. 4c (i)). There could also be an indirect effect if direct target RNAs of the DCL2-dependent silencing encode activators of other genes: the downstream genes would all be upregulated in the mutant (Fig. 4c (ii)). Downregulation of gene expression in the mutant would occur indirectly if a DCL2-dependent sRNA targets mRNAs encoding repressors of gene expression (Fig. 4c (iii)). Correspondingly, the changes to the gene expression in the *lyc* × *pen* hybrids by competition or antagonism of parental DCL2-dependent sRNAs (Fig. 4c) would be a combination of direct and indirect effects, as in *dcl2.*

The 22 nt + DESL represent a subset of the total EPRVs in the tomato genome (Additional file 1: Fig. S4), and to account for the sRNA increase, it is likely that they are transcribed at a higher level following the *lyc* × *pen* hybridization. None of these loci, however, is an intact EPRV, and they are likely fragments of viral genomes that infected ancestors of the modern *Solanum* species [25, 26] with retained features that link them with the DCL2 antiviral RNA silencing pathway [18]. These loci could be associated with the changes to DNA methylation or histone modifications in the hybrids, association with different classes of transposon, or the production of extrachromosomal circular DNA, as with activation of petunia and banana EPRVs [27, 28].

With the involvement of 22-nt sRNAs in hybrids, there is the potential for more complex and far-reaching changes to gene expression than with other size classes of sRNA. The 22-nt size class is specifically associated with translational regulation [29], and it also has the potential to trigger secondary sRNAs that target multiple mRNAs [30, 31]. There is potential for similar outcomes in the many other species carrying EPRVs including bitter orange, rice, lucky bamboo, dahlia, pineapple, grapes, poplar, and fig [25]. In sugar beets [32] and soybeans [33], the EPRVs are associated with 22-nt sRNAs as in *Solanum*. This sRNA-based mechanism involving EPRVs could modify the gene expression in hybrid populations of many species and increase the range of traits available for selection in natural evolution or breeding for agriculture.

## Conclusions

The phenotypes of hybrid plants are influenced partially by non-parental levels of expression in a subset of genes. Here, we show that the non-parental gene expression profile in *lyc* × *pen* hybrids overlaps substantially with the effects of a *dcl2* mutation. Coinciding with this effect, there is a modified profile of DCL2-dependent sRNAs that are predominantly 22 nt in length and associated with endogenous pararetroviruses. We hypothesize that the upregulated sRNAs in the hybrids compete or antagonize the pattern of DCL2-dependent sRNA silencing in the parental plant so that the change in gene expression overlaps with that in a *dcl2* mutant, as observed. These findings provide novel molecular handles on the mechanisms in hybrid *Solanum* that could be relevant to other species*.*

## Methods

### Plant material

Parental lines (tomato (*Solanum lycopersicum*) cv. M82 and *Solanum pennellii* LA716) and hybrid population derived from the crosses were grown from seeds in compost (Levington M3) and maintained in the Botanical Garden Greenhouses (24–18 °C, 16 h/8 h day/night regime) propagated by cuttings so that hybrids and parents could be sampled simultaneously. The only requirement for plant selection was the production of fertile offspring. A replica of the collection was maintained at NIAB (Cambridge, UK). Samples for library preparation were collected for parent and progeny the same day for a period of 2 h. Each sample is a pool of 3–5 young leaves (1 to 3 cm). To compare the plants along the generations for the phenotypic analysis, we grew 4 axillary cuttings per plant for 8 weeks. Dry weight was calculated by weighting the areal part of each plant after being dried in an oven at 65 °C for 1 week.

### RNA extraction and library preparation (mRNA-Seq and sRNA-Seq)

#### RNA-Seq

Five micrograms of total TRIzol-purified RNA purified was treated with Turbo DNase free kit (Thermo Fisher), ribosomal depleted using Ribo-Zero Kit (Epicentre), and libraries prepared using ScriptSeq v2 RNA-Seq Library Preparation Kit (Epicentre), PCR amplified in 9 cycles. In total, 12 libraries were prepared, containing two biological replicates for each of the plants. Equimolecular pooled libraries were sequenced with the Illumina High Output Kit v2 150PE in a NextSeq 550 benchtop machine (Illumina) at the SLCU (UK).

#### sRNA-Seq

One microgram of total TRIzol-purified RNA purified was used for the library preparation using the NEBNext Multiplex Small RNA Library Prep Set for Illumina (NEB) with libraries indexed in 12 cycles PCR. Size selection was performed using BluePippin 3% agarose cassettes (Sage Science). Equimolecular pooled libraries were sequenced with the Illumina High Output Kit v2 30 SE in a NextSeq 550 benchtop machine (Illumina) at the SLCU (UK). RNA was independently isolated for RNA-Seq and sRNA libraries.

### Reference genome and annotation

Reference genome consists of a merged genome where genome assemblies for both parental strains, *Solanum pennellii* and *Solanum lycopersicum* cv. Heinz [34] assembly version SL3.0, were combined into one reference, also including mitochondrial and chloroplast genomes. A merged genome was used to be able to unambiguously identify the origins of NGS reads when mapping in a unique fashion (as described below). A hybrid genome would however have no impact on a multi-mapping strategy in which case the genotype ensures the appropriate origin. The merged genome was sliced into 200 bp adjacent non-overlapping bins. Bins were used as intervals as a basis to count various features which either overlap (such as gene or TE annotation) or map into them (e.g., sRNAs/RNA-Seq reads). Gene annotation and transposable elements are based on ITAG3.2 (“gene_models” and “REPET_repeats_aggressive”) (https://solgenomics.net). MicroRNA annotation was obtained from miRBase release 22.1 [35]. Since precursor coordinates were only available for Heinz assembly version 2.50, we performed a lift over to Heinz assembly 3.0 using Liftoff version 1.6.1 [36].

### Genotyping using RNA-Seq reads

To genotype each F4, we used the SNP information in the RNA-Seq reads [15] for actively transcribed regions. To determine SNPs that differentiate between the two parents, we performed a whole-genome alignment using Mummer v3.23 [37]. As the first step, the subprogram “nucmer” with default settings was used to perform a genome-wide alignment between both assemblies. The delta results were then filtered with “delta-filter -r -q” to exclude chance and repeat induced alignments, leaving only the “best” alignments between both assemblies (only SNPs on the *S. lycopersicum* genome that exhibit homologous alignments from *S. pennellii* and vice versa), resulting in 180,868 alignment blocks. Those were used as a basis to extract SNP positions using “show-snps –CTr” resulting in 23,528,724 SNPs which positions were available for both assemblies.

Mapped RNA-Seq reads (mapping was performed only on one assembly at a time to ensure that homologous genes map to the same coordinates to allow genotyping) were processed using “bcftools mpileup -d10000 -Ov -R $snps -o ${bam}.R.vcf -f $ref ${bam}.bam” for all sequencing libraries to generate RNA-Seq genotype information. The SNP positions (focusing on the *S. lycopersicum* genome) were then used to determine the genotype for each SNP by comparing the RNA-Seq-derived SNPs with the expected SNP derived from the mummer analysis above using the R package “vcfR” and custom scripts. For each mummer SNP, if RNA-Seq information supported only the expected *S. lycopersicum*/*S. pennellii* genotype, this SNP would be annotated as *S. lycopersicum*/*S. pennellii* homozygous transcribed region. If both variants occur, it was annotated as a heterozygous transcribed region. Since this process can be error-prone and we expected the same genotype over larger regions [38], we constructed a hidden Markov model (HMM) using the R package “HMM” to construct a genome-wide genotype map. The assumption was that genotype patterns are relatively large (typically mega bases) containing multiple regions with transcriptional activity, i.e., the genotype of an interval between to transcriptional location of the same genotype is likely to be of the same genotype. Since this process is subject to noise and a genotype section is expected to be large, the HMM Viterbi algorithm was parameterized to account for this using 3 states (homozygous *S. lycopersicum*, homozygous *S. pennellii*, and heterozygous) with respective probabilities (0.45, 0.45, 0.1). Transition probabilities between states were chosen to be very small (1e − 9). This map also allows to infer the genotype of non-RNA-Seq-covered sections. We tested this approach in our parental lines, obtaining a prediction of 99.6% *lyc* homozygosity for *S. lycopersicum* and 98.6% *pen* homozygosity for *S. pennellii* (Fig. 1b).

### Small RNA sequencing data processing and analysis

Small RNAs reads [17] (fastq format) were subjected to 3′ adaptor removal (trimming) using cutadapt v3.1 removing Illumina universal adapters. Sequences with < 15 nt and > 40 nt in length were discarded, and the remaining sequences were mapped to the reference genome (the merged genome as described above). Specifically, mapping was performed using Bowtie version 1.2 with 2 different approaches. Firstly, keeping multi mapping reads with parameters “bowtie –wrapper basic-0 -v 0 -k 1 -m 50 –best -q” which randomly assigns reads to a mapping location among the multiple locations and secondly only keeping uniquely mapping reads with “bowtie –wrapper basic-0 -v 0 -k 1 -m 1 –best –q.” Both were performed requiring 0 mismatches (-v 0). For both approaches, mapped sRNAs reads were counted separately for each library based on overlapping bins as defined above. An additional read counting was also performed for each small RNA size class (20–25 nt length) for each bin for a more detailed analysis. Differential expression (DE) analysis was performed using the R/bioconductor package edgeR with first conduction “glmFit” to fit genewise negative binomial followed by “glmLRT” which conducts likelihood ratio tests. Test intervals were bins as and TMM normalization method was used for “calcNormFactors” library normalization. Comparisons were performed between parents and F4 libraries separately. Multiple test correction was carried calculating the false discovery rate (FDR) from *p*-values using the Benjamini and Hochberg method as implemented in the R function “p.adjust.”

Bins were grouped based on FDR threshold into non-DESL (FDR > 0.9), (0.05 < FDR < 0.9) and DESL (differentially expressed small RNA locus; FDR < 0.05). DESL were further classed into + DESL (F4 > WT) and − DESL (F4 < WT). Bins with insufficient coverage (edgeR default) were excluded from statistical analysis and assigned “none” class. For miRNA analysis, sRNAs reads mapped to our miRNA annotation were normalized to total number of reads in each library.

### RNA sequencing processing and analysis

RNA-Seq libraries [15] were trimmed using cutadapt v3.1 and mapped using STAR version 2.7.5c using “–outFilterMultimapNmax 20 –alignSJoverhangMin 8 –alignIntronMin 20 –alignIntronMax 10,000 –bamRemoveDuplicatesType UniqueIdentical –outFilterMismatchNmax 20” as parameters against the merged reference genome described above. Quantification of gene expression quantification was performed using the R/bioconductor “Rsubread” package. Differential expression (DE) analysis was performed using the R/bioconductor package edgeR using genes as testing intervals and TMM normalization method. Comparisons were performed between the homozygous transcribed genes for each F4 and the corresponding parent separately. Multiple test correction was carried calculating the false discovery rate (FDR) from *p*-values using the Benjamini and Hochberg method as implemented in the R function “p.adjust.” Genes were grouped based on FDR threshold into non-DEG (FDR > 0.9), (0.05 < FDR < 0.9), and DEG (differentially expressed smallRNA locus; FDR < 0.05). DESL were further classed into + DESL (F4 > WT) and − DESL (F4 < WT). Genes with insufficient coverage (edgeR default) were excluded from statistical analysis and assigned “none” class.

### External *dcl2* library re-analysis

#### sRNA-Seq

Previously published raw sRNAs libraries [18, 21] (WT = SRR6436051, SRR6436050 and *dcl2*_mutant = SRR6436048, SRR6436055) were pre-processed the same way as described for the our sRNA-Seq libraries [39]. The adapter sequence used for trimming was found to be “AGATCGGAAGAGCAC” (Illumina adapter). WT libraries were compared against *dcl2* mutant libraries using the same threshold as our sRNA analysis. Similarly, bins were classified into − D2SL for *dcl2* < WT and + D2SL for *dcl2* > WT.

#### RNA-Seq

Previously published raw RNA-Seq libraries [20, 22] (WT = SRR6866906, SRR6866908, SRR6866909 and *dcl2*_mutant = SRR6868335, SRR6868336, SRR6868333) were pre-processed as described previously [39]. WT libraries were compared against *dcl2* mutant libraries using the same threshold as our RNA-Seq analysis. Similarly, genes were classified into − D2G for *dcl2* < WT and + D2G for *dcl2* > WT.

### Phylogenetic analysis

In order to perform an unbiased phylogenetic analysis on EPRV domains for both *S. lycopersicum* and *S. pennellii*, we retrieved consensus nucleic acid sequences encoding for env, inclusion body, movement protein, and polymerase from GIRI repbase (LycEPRV_I) and aligned them to both genome assemblies using BLAT version 36 using with following parameters “-minScore = 25 -minIdentity = 80 -noHead -maxIntron = 1000.” For each domain, BLAT alignments were imported into R using the “hiReadsProcessor” library, and mapping locations were overlapped (based on genomic location) with available annotations as well as bins which come with their respective annotations (again for both genomes). The underlying genomic sequences were written into fasta files for each location encoding annotation in the naming header. All sequences found for a specific domain (env, inclusion body, movement protein, and polymerase) were multiple aligned with “t_coffee” version 13.41.0.28bdc39 using default parameters.

Resulting multiple alignments were subjected to trimAl version 1.4 rev15 to remove gaps (usging “-gappyout”), spurious sequences, or poorly aligned regions. Cleaned up alignments were used to build a phylogentic tree using RAxML-NG version 1.0.1, a phylogenetic tree inference tool which uses maximum-likelihood (ML) optimality criterion (“raxml-ng –msa $aln –model GTR + G”). Phylogenetic tree visualization of resulting trees was performed using FigTree version 1.4.4.

### Data visualization and statistical analysis

Plots were carried out using ggplot2 [40]. Statistical tests were carried out in R version 4.0. Pearson correlation was calculated using “cor.test” R function with default parameters (two-sided).

## Supplementary Information

**Additional file 1:** **Fig. S1.** Phenotyping analysisof the hybrid population along the generations. **Fig. S2.** F4 genomes are chimeras of *S. lycopersicum* and *S. pennellii*. **Fig. S3.** Gene expression andsRNA analysis for the S pennellii homozygous transcribed regions for each F4compared to *pen*. **Fig. S4.** Phylogenetic relationship of EPRV envelopesequence.**Fig. S5.** Size distribution of sRNA loci in the F4s. **Fig. S6.** Heatmap of the inheritance of DESLs in each family. **Fig. S7.** Differentiallyexpressed genes are shared between the F4s and *dcl2* mutant.

**Additional file 2:** **Table S1.** RNAseq processed data. List of genesidentified as differentially expressed, “in between”, or non-DEG for each ofthe F4 plants and dcl2 mutant. DCL genes expression data in F4s has beenextracted and shown in last sheet.

**Additional file 3:** **Table S2.** sRNAseq processed data. List of sRNA lociclassified as differentially expressed, “in between”, or non-DESL for each ofthe F4 plants and dcl2 mutant. miRNAs normalised counts in parents and F4s,with bar plot corresponding to miR6026.

**Additional file 4**: Reviewhistory.

## Data Availability

Biological material is available by request to the corresponding author (dcb40@cam.ac.uk). The datasets supporting the conclusions of this article are available in the ArrayExpress repository (https://www.ebi.ac.uk/arrayexpress); smallRNA-Seq (sRNA-Seq data from the F4s and parental lines) [17]: E-MTAB-10613; RNA-S (RNA-Seq data from the F4s and parental lines) [15]: E-MTAB-10660; and NCBI for sRNAs of WT and *dcl2* [18, 21] and RNA-Seq of WT and *dcl2* [20, 22]. RNA-Seq and sRNA-Seq processed data are available as Additional file 2: Table S1 and Additional file 3: Table S2. Computationally reproducible scripts are available under a Creative Commons Attribution 4.0 International License at Zenodo 10.5281/zenodo.6477339 [39]. The scripts are also available under Creative Commons Attribution 4.0 International License at GitHub: https://github.com/seb-mueller/scripts_tribe [41].
