# Supplementary material for: Interspecific hybridization in tomato influences endogenous viral sRNAs and alters gene expression
Source: Genome Biol. 2022 May 21;23:120. doi: 10.1186/s13059-022-02685-z (PMC9124383; doi:10.1186/s13059-022-02685-z)
Supplement: Supplementary file 1 — Additional file 1: Fig. S1. Phenotyping analysisof the hybrid population along the generations. Fig. S2. F4 genomes are chimeras of S. lycopersicum and S. pennellii. Fig. S3. Gene expression andsRNA analysis for the S pennellii homozygous transcribed regions for each F4compared to pen. Fig. S4. Phylogenetic relationship of EPRV envelopesequence.Fig. S5. Size distribution of sRNA loci in the F4s. Fig. S6. Heatmap of the inheritance of DESLs in each family. Fig. S7. Differentiallyexpressed genes are shared between the F4s and dcl2 mutant. [file 13059_2022_2685_MOESM1_ESM.pdf]

Interspecific hybridization in tomato influences endogenous viral sRNAs and alters gene expression

Sara Lopez-Gomollon, Sebastian Y. Müller & David C. Baulcombe*.

Additional file 1: Figures S1-S7

Fig. S1. Phenotyping analysis of the hybrid population along the generations. For the phenotypic study, 4 cuttings (obtained from axillary new leaves) per plant were grown simultaneously for 8 weeks**.** **a.** The 5th leaf from the bottom was used for the comparative phenotypic analysis. All pictures at the same scale being scale bar 10cm. **b.** Quantitative analysis of phenotypical traits (dry weight, height, and number of leaves). Parental lines in red, F1-F3 plants in light blue and F4 plants in dark blue. Dotted lines show the values for the parental lines for comparison to identify transgenic phenotypes. Box plots elements: box limits, upper and lower quartiles; center line, median; whiskers, 1.5x interquartile range; points, outliers.

Fig.S2. F4 genomes are chimeras of *S. lycopersicum* and *S. pennellii*. RNAseq-based SNP genotyping for each of the F4 plants (P1512, P2561, P2562, P3611, P3612, P4041, P4042). Top track (within each chromosome panel): For each tomato nuclear chromosome is it shown the genotypic results (red: region homozygous transcribed for *S. lycopersicum*, green: heterozygous transcribed, light blue: homozygous transcribed for *S. pennellii*, pink: could not be determined). Bottom track: chromatin status divided into euchromatin (light grey) or heterochromatin (dark grey) as described [14]. As expected, there were very few meiotic crossovers in the pericentromeric region. Preferential inheritance of some regions of the genomes including those that were homozygous transcribed *lyc* in all F4 plants (regions in chromosomes 2,8,10 and 11) may be due to selection against genes or combinations of genes that caused failure to produce progeny in some F2 and F3 progeny.

Fig.S3. Gene expression and sRNA analysis for the S pennellii homozygous transcribed regions for each F4 compared to *pen*. a. Percentage of genes in each group for the F4s compared to *pen* parent, calculated from the total number of genes in the *pen* homozygous transcribed region, being each bar an individual plant. The right-hand panel shows an expanded view of the +DEG (red) and -

DEG (blue). **b.** Percentage of sRNA loci in each group, calculated from the total number of sRNA loci in the *pen* homozygous transcribed region. Groups are sRNA loci upregulated (+DESL, red), downregulated (-DESL, blue), "in between" (lilac) and non-differentially expressed (non-DESL, yellow). The right-hand panel shows an expanded view of the +DESL (red) and -DESL (blue). Thresholds: DESL, FDR <0.05; "in between" DESL, 0.05 <FDR < 0.9; non-DESL, FDR > 0.9. **c-e.** Box plot showing the percentage of *pen* sRNA loci (**c**) genome wide, (**d**) to each genomic feature, (**e**) to TE order, that are +DESL or -DESL. Ratio of +DESL/-DESL is shown at the bottom. Each plant is represented by different dot shapes. Box plots elements: box limits, upper and lower quartiles; center line, median; whiskers, from each quartile to the minimum or maximum. For comparison, colored lines are the percentage values for +DESL (red) and -DESL (blue) mapping the whole genome (obtained from **c**).

Fig.S4. Phylogenetic relationship of EPRV envelope sequence. Polar Tree layout of the phylogenetic tree build based on envelope sequences found on *S. lycopersicum* by manual BLAT search using as original the envelope sequence from EPRV on GIRI, followed by RaxML tree construction. Node names consist of domain name (envelope), followed by coordinates on genome (chromosome name and start coordinate), overlapping transposon family according to REPET annotation (NA if no overlap), and overlapping type of sRNA loci highlighting +DESL in red and -DESL in blue.

Fig.S5. Size distribution of sRNA loci in the F4s. Name of plant shown at the top of each set of panels. Normalised sRNA counts plotted by size for -DESL, +DESL and non-DESL **(a)** genome wide and **(b)** mapping to each TE order. **c.** Normalised sRNA counts plotted by size in each F4 (-DESL, +DESL and non-DESL) overlapping with sRNA loci in *dcl2* (-D2SL, +D2SL and non-D2SL). Red line- sRNA counts for *lyc*, blue line -sRNA counts for each F4. Solid line- counts for all sRNA loci in each group category for that plant. Dotted line – counts for sRNA in each category that map to EPRVs.

Fig.S6. Heatmap of the inheritance of DESLs in each family. Normalised reads for each generation for the sRNA loci identified as *lyc* DESL in one of the F4s per family (in bold letter on top), filtered for |FC| >9 and FDR < 1e-10. The strict threshold was used to reduce the size of the plot by focusing on the most significant changes. Normalization was performed for each bin by centering and scaling (z-

score) across the lineages (rows). The names of bins (rows) correspond to the genomic coordinates of their first base. Annotation tracks were derived by overlapping the respective bins with genomic annotation features. Columns correspond to data for each plant (in black) and the generation that they belong to (in red on top of the name of the plant).

Fig.S7. Differentially expressed genes are shared between the F4s and *dcl2* mutant. a. Venn diagram of genes upregulated (+DEG) and downregulated (-DEG) for an F4 plant compared to the genes upregulated (+D2G) or downregulated (-D2G) in the *dcl2* mutant. Statistical association determined by Fisher's Exact Test, ** two-tailed p <10e-6, *** two-tailed p <2.2e-16. **b.** Scatter plot of the gene expression log fold change (FC) for each F4 compared to the *dcl2* mutant. Grey: non-DEG or in between for any of the two plants; green: DEG for the F4 or *dcl2*; purple: DEG for F4 and *dcl2*. Pearson Correlation coefficient values (r) for the DEG and D2G genes (purple genes) for each scatter plot, p = p value.

Figure S1

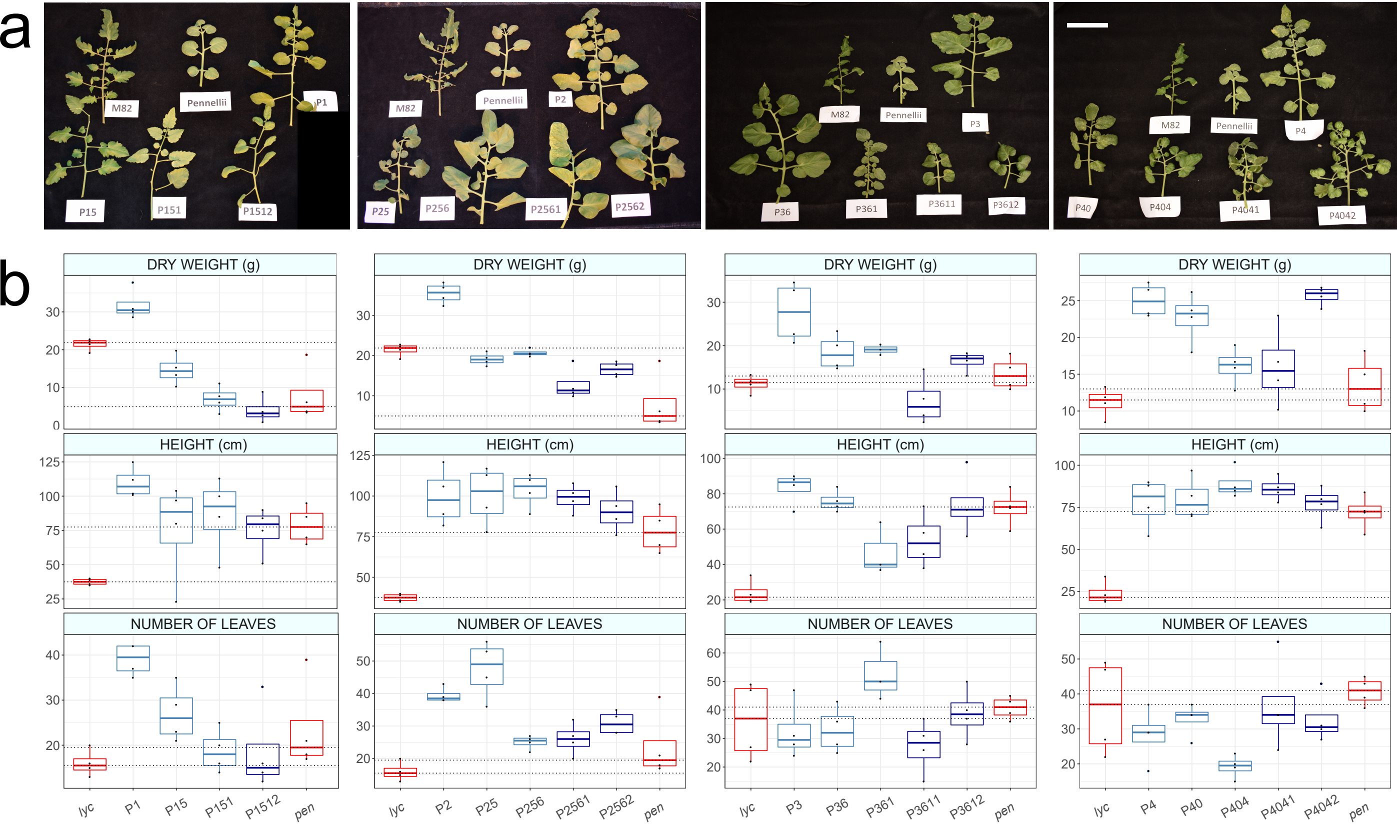

a
M82
Pennellii
P1
P15
P151
P1512
M82
Pennellii
P2
P25
P256
P2561
P2562
M82
Pennellii
P3
P36
P361
P3611
P3612
M82
Pennellii
P4
P40
P404
P4041
P4042
b
DRY WEIGHT (g)
HEIGHT (cm)
NUMBER OF LEAVES
lyc
P1
P15
P151
P1512
pen
lyc
P2
P25
P256
P2561
P2562
pen
lyc
P3
P36
P361
P3611
P3612
pen
lyc
P4
P40
P404
P4041
P4042
pen

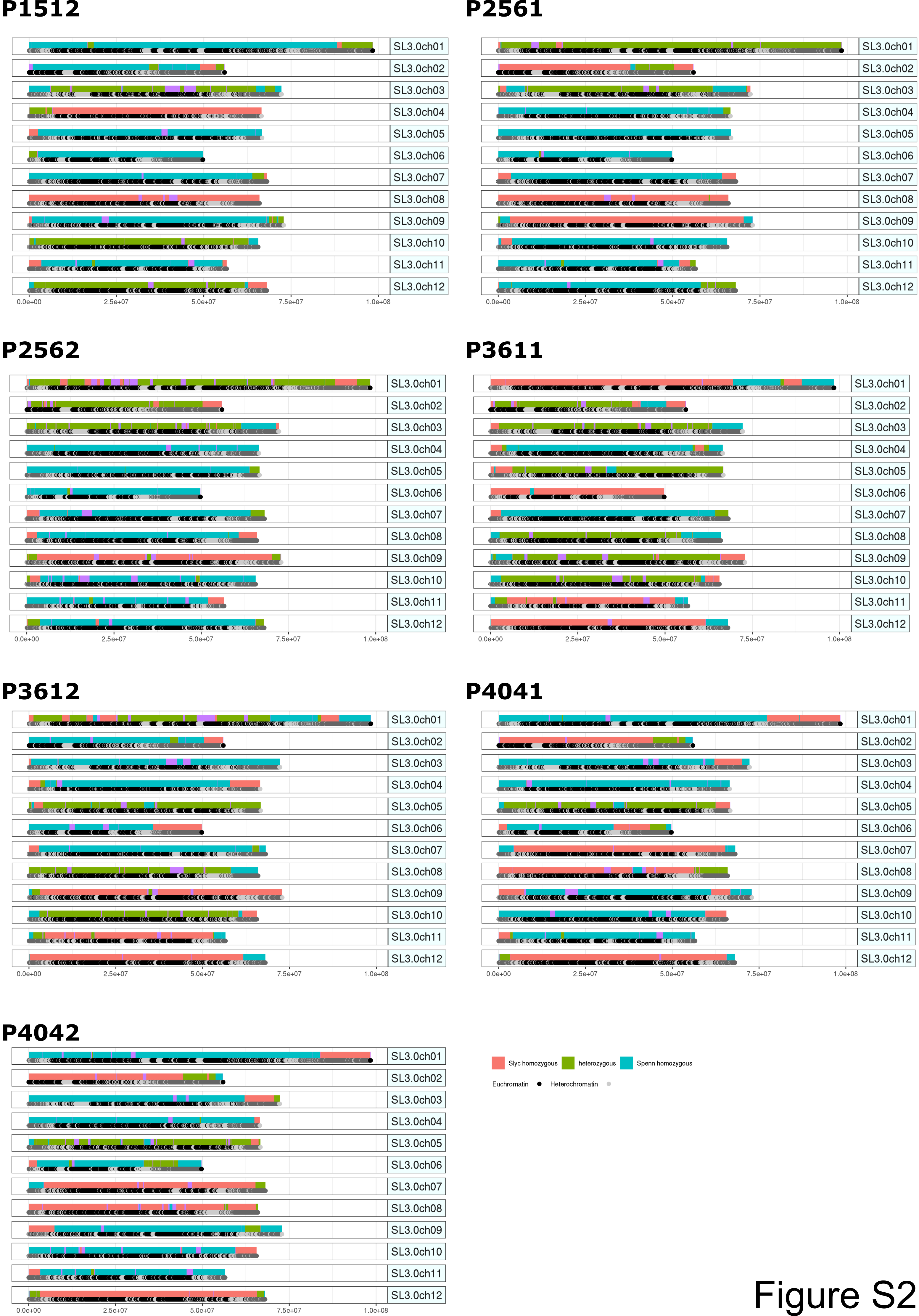
P1512
SL3.0ch01
SL3.0ch02
SL3.0ch03
SL3.0ch04
SL3.0ch05
SL3.0ch06
SL3.0ch07
SL3.0ch08
SL3.0ch09
SL3.0ch10
SL3.0ch11
SL3.0ch12
0.0e+00
2.5e+07
5.0e+07
7.5e+07
1.0e+08
P2561
SL3.0ch01
SL3.0ch02
SL3.0ch03
SL3.0ch04
SL3.0ch05
SL3.0ch06
SL3.0ch07
SL3.0ch08
SL3.0ch09
SL3.0ch10
SL3.0ch11
SL3.0ch12
0.0e+00
2.5e+07
5.0e+07
7.5e+07
1.0e+08
P2562
SL3.0ch01
SL3.0ch02
SL3.0ch03
SL3.0ch04
SL3.0ch05
SL3.0ch06
SL3.0ch07
SL3.0ch08
SL3.0ch09
SL3.0ch10
SL3.0ch11
SL3.0ch12
0.0e+00
2.5e+07
5.0e+07
7.5e+07
1.0e+08
P3611
SL3.0ch01
SL3.0ch02
SL3.0ch03
SL3.0ch04
SL3.0ch05
SL3.0ch06
SL3.0ch07
SL3.0ch08
SL3.0ch09
SL3.0ch10
SL3.0ch11
SL3.0ch12
0.0e+00
2.5e+07
5.0e+07
7.5e+07
1.0e+08
P3612
SL3.0ch01
SL3.0ch02
SL3.0ch03
SL3.0ch04
SL3.0ch05
SL3.0ch06
SL3.0ch07
SL3.0ch08
SL3.0ch09
SL3.0ch10
SL3.0ch11
SL3.0ch12
0.0e+00
2.5e+07
5.0e+07
7.5e+07
1.0e+08
P4041
SL3.0ch01
SL3.0ch02
SL3.0ch03
SL3.0ch04
SL3.0ch05
SL3.0ch06
SL3.0ch07
SL3.0ch08
SL3.0ch09
SL3.0ch10
SL3.0ch11
SL3.0ch12
0.0e+00
2.5e+07
5.0e+07
7.5e+07
1.0e+08
P4042
SL3.0ch01
SL3.0ch02
SL3.0ch03
SL3.0ch04
SL3.0ch05
SL3.0ch06
SL3.0ch07
SL3.0ch08
SL3.0ch09
SL3.0ch10
SL3.0ch11
SL3.0ch12
0.0e+00
2.5e+07
5.0e+07
7.5e+07
1.0e+08
Slyc homozygous
heterozygous
Spenn homozygous
Euchromatin
Heterochromatin
Figure S2

Figure S3

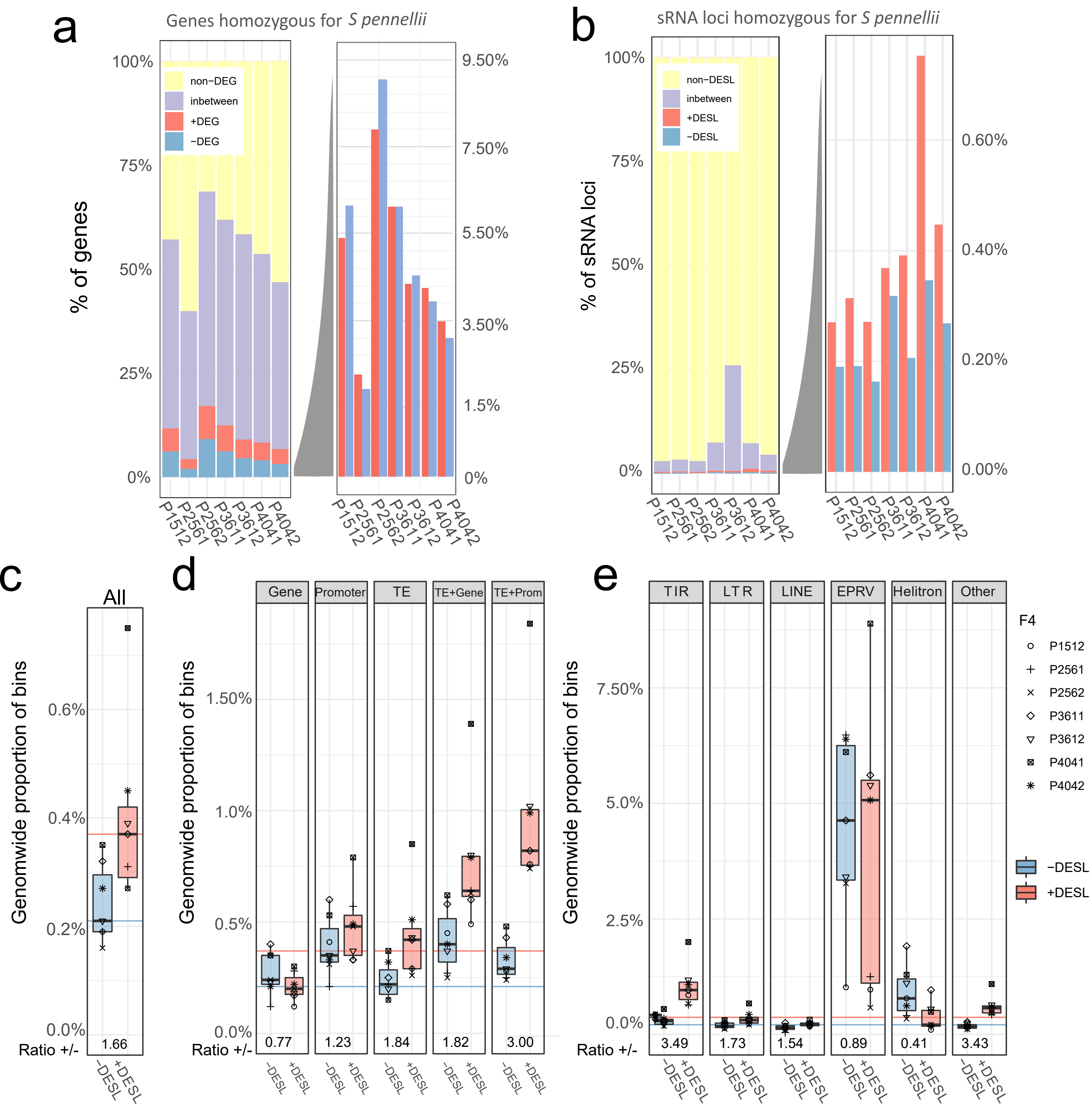

a
Genes homozygous for S pennellii
% of genes
non−DEG
inbetween
+DEG
−DEG
100%
75%
50%
25%
0%
9.50%
7.50%
5.50%
3.50%
1.5%
0%
P1512
P2561
P2562
P3611
P3612
P4041
P4042
b
sRNA loci homozygous for S pennellii
% of sRNA loci
non−DESL
inbetween
+DESL
−DESL
0.60%
0.40%
0.20%
0.00%
c
All
Genomwide proportion of bins
Ratio +/-
1.66
−DESL
+DESL
d
Gene
Promoter
TE
TE+Gene
TE+Prom
1.50%
1.0%
0.5%
0.0%
0.6%
0.4%
0.2%
0.77
1.23
1.84
1.82
3.00
e
TIR
LTR
LINE
EPRV
Helitron
Other
7.50%
5.0%
2.5%
3.49
1.73
1.54
0.89
0.41
3.43
F4

Figure S4

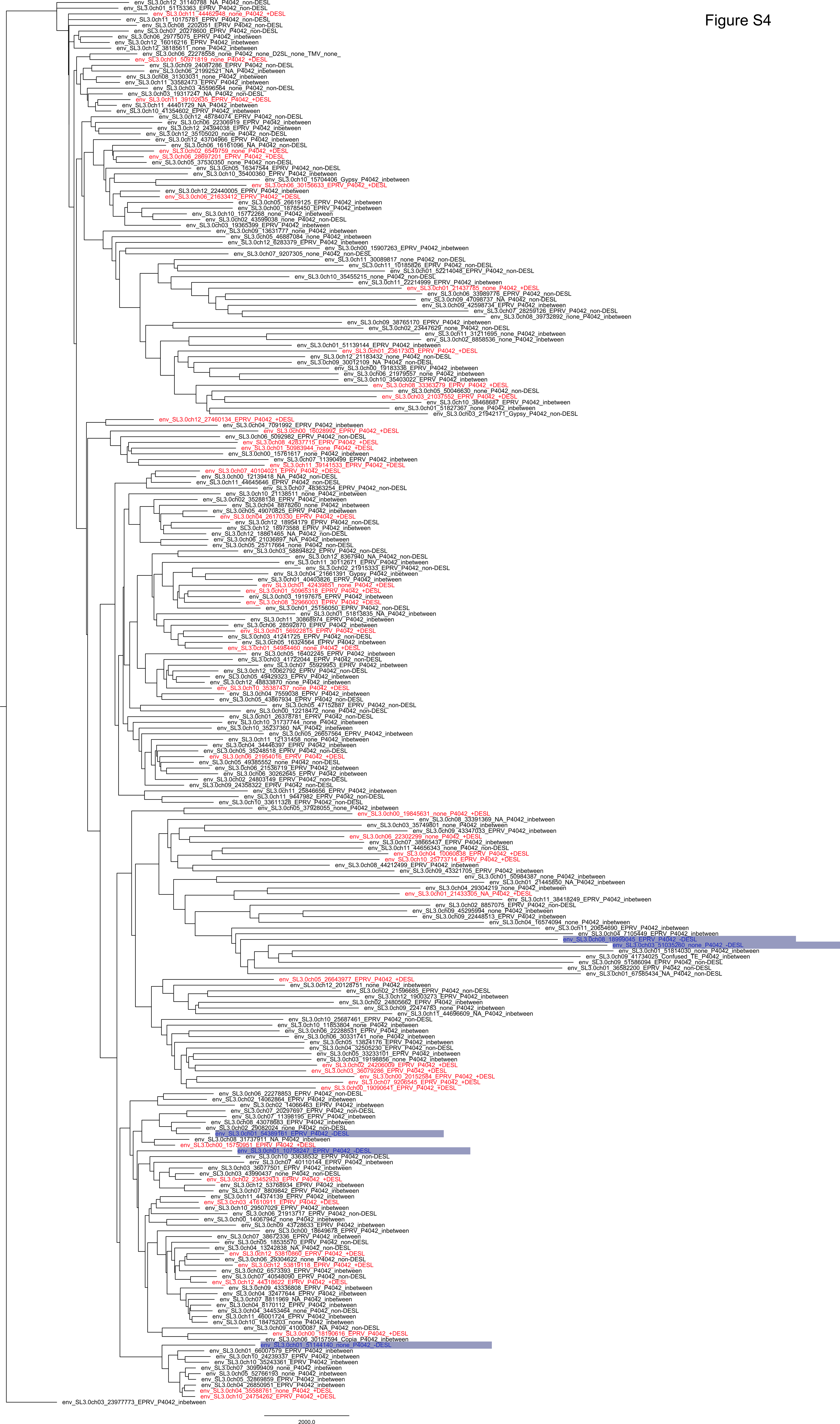
env_SL3.0ch12_31140788_NA_P4042_non-DESL
env_SL3.0ch01_51153363_EPRV_P4042_non-DESL
env_SL3.0ch11_44462948_none_P4042_+DESL
env_SL3.0ch11_10175781_EPRV_P4042_non-DESL
env_SL3.0ch08_2202051_EPRV_P4042_non-DESL
env_SL3.0ch07_20278600_EPRV_P4042_non-DESL
env_SL3.0ch06_29775075_EPRV_P4042_inbetween
env_SL3.0ch12_16016216_EPRV_P4042_inbetween
env_SL3.0ch12_38185611_none_P4042_inbetween
env_SL3.0ch06_22278558_none_P4042_none_D2SL_none_TMV_none_
env_SL3.0ch01_50971819_none_P4042_+DESL
env_SL3.0ch09_24087286_EPRV_P4042_non-DESL
env_SL3.0ch06_21992521_NA_P4042_inbetween
env_SL3.0ch08_31303031_none_P4042_inbetween
env_SL3.0ch11_33582473_EPRV_P4042_inbetween
env_SL3.0ch03_45596564_none_P4042_non-DESL
env_SL3.0ch03_19317247_NA_P4042_non-DESL
env_SL3.0ch11_39102635_EPRV_P4042_+DESL
env_SL3.0ch11_44401729_NA_P4042_inbetween
env_SL3.0ch10_41354602_EPRV_P4042_inbetween
env_SL3.0ch12_48784074_EPRV_P4042_non-DESL
env_SL3.0ch06_22306919_EPRV_P4042_inbetween
env_SL3.0ch12_24394038_EPRV_P4042_inbetween
env_SL3.0ch12_35105020_none_P4042_non-DESL
env_SL3.0ch12_43704966_EPRV_P4042_inbetween
env_SL3.0ch06_16161096_NA_P4042_non-DESL
env_SL3.0ch02_6549759_none_P4042_+DESL
env_SL3.0ch06_28697201_EPRV_P4042_+DESL
env_SL3.0ch05_37530350_none_P4042_non-DESL
env_SL3.0ch05_16347544_EPRV_P4042_non-DESL
env_SL3.0ch10_35400360_EPRV_P4042_inbetween
env_SL3.0ch10_15704406_Gypsy_P4042_inbetween
env_SL3.0ch06_30156633_EPRV_P4042_+DESL
env_SL3.0ch12_22440005_EPRV_P4042_inbetween
env_SL3.0ch06_21633412_EPRV_P4042_+DESL
env_SL3.0ch05_26619125_EPRV_P4042_inbetween
env_SL3.0ch00_18785450_EPRV_P4042_inbetween
env_SL3.0ch10_15772268_none_P4042_inbetween
env_SL3.0ch02_43599038_none_P4042_non-DESL
env_SL3.0ch03_19365399_EPRV_P4042_inbetween
env_SL3.0ch09_13631777_none_P4042_inbetween
env_SL3.0ch05_46887084_none_P4042_inbetween
env_SL3.0ch12_6283379_EPRV_P4042_inbetween
env_SL3.0ch00_15907263_EPRV_P4042_inbetween
env_SL3.0ch07_9207305_none_P4042_non-DESL
env_SL3.0ch11_30089817_none_P4042_non-DESL
env_SL3.0ch11_10185826_EPRV_P4042_non-DESL
env_SL3.0ch01_52214048_EPRV_P4042_non-DESL
env_SL3.0ch10_35455215_none_P4042_non-DESL
env_SL3.0ch11_22214999_EPRV_P4042_inbetween
env_SL3.0ch01_21437785_none_P4042_+DESL
env_SL3.0ch06_33989776_EPRV_P4042_non-DESL
env_SL3.0ch09_47098737_NA_P4042_non-DESL
env_SL3.0ch09_42598734_EPRV_P4042_inbetween
env_SL3.0ch07_28259126_EPRV_P4042_non-DESL
env_SL3.0ch08_39732892_none_P4042_inbetween
env_SL3.0ch09_38765170_EPRV_P4042_inbetween
env_SL3.0ch02_23447629_none_P4042_non-DESL
env_SL3.0ch11_31211695_none_P4042_inbetween
env_SL3.0ch02_8858536_none_P4042_inbetween
env_SL3.0ch01_51139144_EPRV_P4042_inbetween
env_SL3.0ch01_23617303_EPRV_P4042_+DESL
env_SL3.0ch12_21183432_none_P4042_non-DESL
env_SL3.0ch09_30012109_NA_P4042_non-DESL
env_SL3.0ch00_19183336_EPRV_P4042_inbetween
env_SL3.0ch06_21979557_none_P4042_inbetween
env_SL3.0ch10_35403022_EPRV_P4042_inbetween
env_SL3.0ch08_33363279_EPRV_P4042_+DESL
env_SL3.0ch05_50046630_none_P4042_non-DESL
env_SL3.0ch03_21037552_EPRV_P4042_+DESL
env_SL3.0ch10_38468687_EPRV_P4042_inbetween
env_SL3.0ch01_51827367_none_P4042_inbetween
env_SL3.0ch03_21942171_Gypsy_P4042_non-DESL
env_SL3.0ch12_27460134_EPRV_P4042_+DESL
env_SL3.0ch04_7091992_EPRV_P4042_inbetween
env_SL3.0ch00_16028992_EPRV_P4042_+DESL
env_SL3.0ch06_5092982_EPRV_P4042_non-DESL
env_SL3.0ch08_42837715_EPRV_P4042_+DESL
env_SL3.0ch01_50983944_none_P4042_+DESL
env_SL3.0ch00_15761617_none_P4042_inbetween
env_SL3.0ch07_11390499_EPRV_P4042_inbetween
env_SL3.0ch11_39141533_EPRV_P4042_+DESL
env_SL3.0ch07_40104021_EPRV_P4042_+DESL
env_SL3.0ch00_12139418_NA_P4042_non-DESL
env_SL3.0ch11_44645646_EPRV_P4042_non-DESL
env_SL3.0ch07_48363254_EPRV_P4042_non-DESL
env_SL3.0ch10_21138511_none_P4042_inbetween
env_SL3.0ch02_35288138_EPRV_P4042_inbetween
env_SL3.0ch04_8878260_none_P4042_inbetween
env_SL3.0ch05_49070825_EPRV_P4042_inbetween
env_SL3.0ch04_26170330_EPRV_P4042_+DESL
env_SL3.0ch12_18954179_EPRV_P4042_non-DESL
env_SL3.0ch12_18973588_EPRV_P4042_inbetween
env_SL3.0ch12_18861465_NA_P4042_non-DESL
env_SL3.0ch06_21036897_NA_P4042_inbetween
env_SL3.0ch05_25717664_none_P4042_non-DESL
env_SL3.0ch03_58894822_EPRV_P4042_non-DESL
env_SL3.0ch12_8367940_NA_P4042_non-DESL
env_SL3.0ch11_30112671_EPRV_P4042_inbetween
env_SL3.0ch02_21915333_EPRV_P4042_non-DESL
env_SL3.0ch04_21661391_Gypsy_P4042_inbetween
env_SL3.0ch01_40403826_EPRV_P4042_inbetween
env_SL3.0ch01_42439851_none_P4042_+DESL
env_SL3.0ch01_50965318_EPRV_P4042_+DESL
env_SL3.0ch03_19197675_EPRV_P4042_inbetween
env_SL3.0ch08_32966003_EPRV_P4042_+DESL
env_SL3.0ch01_25156050_EPRV_P4042_non-DESL
env_SL3.0ch01_51813835_NA_P4042_inbetween
env_SL3.0ch11_30868974_EPRV_P4042_inbetween
env_SL3.0ch06_28592870_EPRV_P4042_inbetween
env_SL3.0ch01_56922815_EPRV_P4042_+DESL
env_SL3.0ch03_41241725_EPRV_P4042_non-DESL
env_SL3.0ch05_16324564_EPRV_P4042_inbetween
env_SL3.0ch01_54984460_none_P4042_+DESL
env_SL3.0ch05_16402245_EPRV_P4042_inbetween
env_SL3.0ch03_41722044_EPRV_P4042_non-DESL
env_SL3.0ch07_55929953_EPRV_P4042_inbetween
env_SL3.0ch12_10062792_EPRV_P4042_non-DESL
env_SL3.0ch05_49429323_EPRV_P4042_inbetween
env_SL3.0ch12_48833870_none_P4042_inbetween
env_SL3.0ch10_35387437_none_P4042_+DESL
env_SL3.0ch04_7559038_EPRV_P4042_inbetween
env_SL3.0ch05_43867934_EPRV_P4042_non-DESL
env_SL3.0ch05_47152887_EPRV_P4042_non-DESL
env_SL3.0ch00_12218472_none_P4042_non-DESL
env_SL3.0ch01_26378781_EPRV_P4042_non-DESL
env_SL3.0ch10_31737744_none_P4042_inbetween
env_SL3.0ch10_35237360_NA_P4042_inbetween
env_SL3.0ch05_26657564_EPRV_P4042_inbetween
env_SL3.0ch11_12131458_none_P4042_inbetween
env_SL3.0ch04_34446397_EPRV_P4042_inbetween
env_SL3.0ch05_35248518_EPRV_P4042_non-DESL
env_SL3.0ch06_21954016_EPRV_P4042_+DESL
env_SL3.0ch05_49385552_none_P4042_non-DESL
env_SL3.0ch06_21536719_EPRV_P4042_inbetween
env_SL3.0ch06_30262645_EPRV_P4042_inbetween
env_SL3.0ch02_24803149_EPRV_P4042_non-DESL
env_SL3.0ch09_24358322_EPRV_P4042_non-DESL
env_SL3.0ch11_25846656_EPRV_P4042_inbetween
env_SL3.0ch11_9447982_EPRV_P4042_non-DESL
env_SL3.0ch10_33611328_EPRV_P4042_non-DESL
env_SL3.0ch05_37928055_none_P4042_inbetween
env_SL3.0ch00_19845631_none_P4042_+DESL
env_SL3.0ch08_33391369_NA_P4042_inbetween
env_SL3.0ch03_35749801_none_P4042_inbetween
env_SL3.0ch09_43347033_EPRV_P4042_inbetween
env_SL3.0ch06_22302299_none_P4042_+DESL
env_SL3.0ch07_38665437_EPRV_P4042_inbetween
env_SL3.0ch11_44656343_none_P4042_non-DESL
env_SL3.0ch04_10060838_EPRV_P4042_+DESL
env_SL3.0ch10_25773714_EPRV_P4042_+DESL
env_SL3.0ch08_44212499_EPRV_P4042_inbetween
env_SL3.0ch09_43321705_EPRV_P4042_inbetween
env_SL3.0ch01_50984387_none_P4042_inbetween
env_SL3.0ch01_21445850_NA_P4042_inbetween
env_SL3.0ch04_29304219_none_P4042_inbetween
env_SL3.0ch01_21433305_NA_P4042_+DESL
env_SL3.0ch11_38418249_EPRV_P4042_inbetween
env_SL3.0ch02_8857075_EPRV_P4042_non-DESL
env_SL3.0ch09_45295994_none_P4042_inbetween
env_SL3.0ch09_22448513_EPRV_P4042_inbetween
env_SL3.0ch04_16574094_none_P4042_inbetween
env_SL3.0ch11_20654690_EPRV_P4042_inbetween
env_SL3.0ch04_7105449_EPRV_P4042_inbetween
env_SL3.0ch08_18999045_EPRV_P4042_-DESL
env_SL3.0ch03_51035260_none_P4042_-DESL
env_SL3.0ch01_51814030_none_P4042_inbetween
env_SL3.0ch09_41734025_Confused_TE_P4042_inbetween
env_SL3.0ch09_51586094_EPRV_P4042_non-DESL
env_SL3.0ch01_36582200_EPRV_P4042_non-DESL
env_SL3.0ch01_67585434_NA_P4042_non-DESL
env_SL3.0ch05_26643977_EPRV_P4042_+DESL
env_SL3.0ch12_20128751_none_P4042_inbetween
env_SL3.0ch02_21596685_EPRV_P4042_non-DESL
env_SL3.0ch12_19003273_EPRV_P4042_inbetween
env_SL3.0ch02_24805662_EPRV_P4042_inbetween
env_SL3.0ch09_22474783_none_P4042_inbetween
env_SL3.0ch11_44696609_NA_P4042_inbetween
env_SL3.0ch10_25687461_EPRV_P4042_non-DESL
env_SL3.0ch10_11853804_none_P4042_inbetween
env_SL3.0ch06_22288531_EPRV_P4042_inbetween
env_SL3.0ch06_30331741_none_P4042_inbetween
env_SL3.0ch05_13824176_EPRV_P4042_inbetween
env_SL3.0ch04_32505230_EPRV_P4042_non-DESL
env_SL3.0ch05_33233101_EPRV_P4042_inbetween
env_SL3.0ch03_19198856_none_P4042_inbetween
env_SL3.0ch02_24206009_EPRV_P4042_+DESL
env_SL3.0ch03_36079286_EPRV_P4042_+DESL
env_SL3.0ch00_20152584_EPRV_P4042_+DESL
env_SL3.0ch07_9206545_EPRV_P4042_+DESL
env_SL3.0ch00_19090641_EPRV_P4042_+DESL
env_SL3.0ch06_22278853_EPRV_P4042_non-DESL
env_SL3.0ch02_14062864_EPRV_P4042_inbetween
env_SL3.0ch02_14066463_EPRV_P4042_inbetween
env_SL3.0ch07_20297697_EPRV_P4042_non-DESL
env_SL3.0ch07_11398195_EPRV_P4042_inbetween
env_SL3.0ch08_43078683_EPRV_P4042_inbetween
env_SL3.0ch02_29082024_none_P4042_non-DESL
env_SL3.0ch01_54389161_EPRV_P4042_-DESL
env_SL3.0ch08_31737911_NA_P4042_inbetween
env_SL3.0ch00_15750951_EPRV_P4042_+DESL
env_SL3.0ch01_10758247_EPRV_P4042_-DESL
env_SL3.0ch10_33638532_EPRV_P4042_non-DESL
env_SL3.0ch07_40110144_EPRV_P4042_inbetween
env_SL3.0ch03_36077501_EPRV_P4042_inbetween
env_SL3.0ch03_43990437_none_P4042_non-DESL
env_SL3.0ch02_23452933_EPRV_P4042_+DESL
env_SL3.0ch12_53768934_EPRV_P4042_inbetween
env_SL3.0ch07_8809842_EPRV_P4042_inbetween
env_SL3.0ch11_44374139_EPRV_P4042_inbetween
env_SL3.0ch03_41610911_EPRV_P4042_+DESL
env_SL3.0ch10_29507029_EPRV_P4042_inbetween
env_SL3.0ch06_21913717_EPRV_P4042_non-DESL
env_SL3.0ch00_14067942_none_P4042_inbetween
env_SL3.0ch09_43728633_EPRV_P4042_inbetween
env_SL3.0ch00_18649678_EPRV_P4042_inbetween
env_SL3.0ch07_38672336_EPRV_P4042_inbetween
env_SL3.0ch05_18535570_EPRV_P4042_non-DESL
env_SL3.0ch04_13242838_NA_P4042_non-DESL
env_SL3.0ch12_53810860_EPRV_P4042_+DESL
env_SL3.0ch06_29304622_none_P4042_non-DESL
env_SL3.0ch12_53819118_EPRV_P4042_+DESL
env_SL3.0ch02_6573393_EPRV_P4042_inbetween
env_SL3.0ch07_40548090_EPRV_P4042_non-DESL
env_SL3.0ch12_44318622_EPRV_P4042_+DESL
env_SL3.0ch09_43336808_EPRV_P4042_inbetween
env_SL3.0ch04_32477644_EPRV_P4042_inbetween
env_SL3.0ch07_8811969_NA_P4042_inbetween
env_SL3.0ch04_8170112_EPRV_P4042_inbetween
env_SL3.0ch04_34453464_none_P4042_non-DESL
env_SL3.0ch11_46001724_EPRV_P4042_inbetween
env_SL3.0ch10_18475203_none_P4042_inbetween
env_SL3.0ch09_41000087_NA_P4042_non-DESL
env_SL3.0ch00_18190616_EPRV_P4042_+DESL
env_SL3.0ch06_30157594_Copia_P4042_inbetween
env_SL3.0ch01_51144140_none_P4042_-DESL
env_SL3.0ch01_66007579_EPRV_P4042_inbetween
env_SL3.0ch10_24239337_EPRV_P4042_inbetween
env_SL3.0ch10_35243361_EPRV_P4042_inbetween
env_SL3.0ch07_30999409_none_P4042_inbetween
env_SL3.0ch05_52766193_none_P4042_inbetween
env_SL3.0ch05_32869859_EPRV_P4042_inbetween
env_SL3.0ch04_26850951_EPRV_P4042_inbetween
env_SL3.0ch04_35588761_none_P4042_+DESL
env_SL3.0ch10_24754262_EPRV_P4042_+DESL
env_SL3.0ch03_23977773_EPRV_P4042_inbetween
2000.0

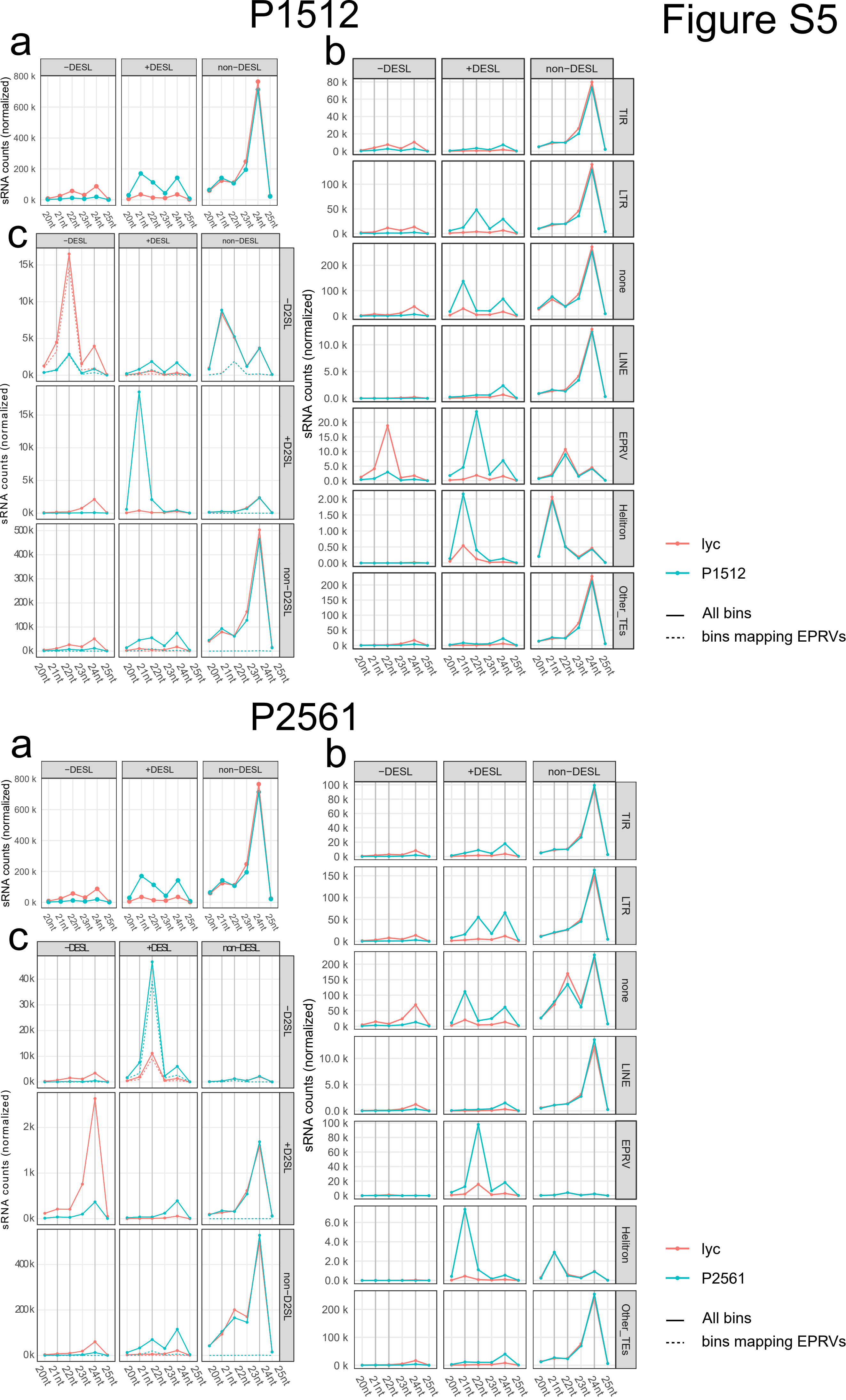

P1512
a
b
c
−DESL
+DESL
non−DESL
−D2SL
+D2SL
non−D2SL
TIR
LTR
none
LINE
EPRV
Helitron
Other_TEs
sRNA counts (normalized)
20nt
21nt
22nt
23nt
24nt
25nt
lyc
P1512
All bins
bins mapping EPRVs
P2561
a
b
c
lyc
P2561
All bins
bins mapping EPRVs

Figure S5

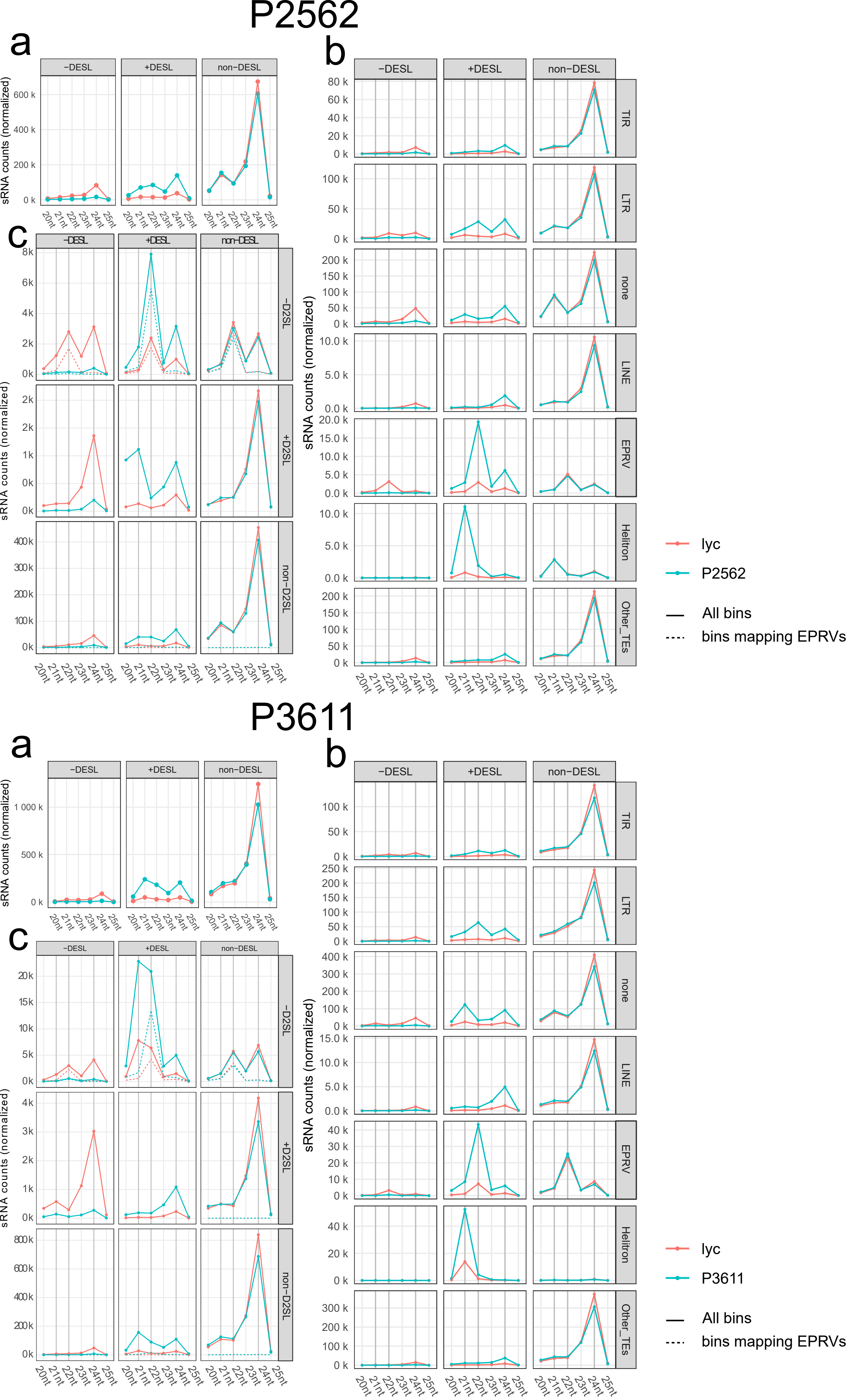

P2562
a
b
c
-DESL
+DESL
non-DESL
sRNA counts (normalized)
-D2SL
+D2SL
non-D2SL
TIR
LTR
none
LINE
EPRV
Helitron
Other_TEs
20nt
21nt
22nt
23nt
24nt
25nt
lyc
P2562
All bins
bins mapping EPRVs
P3611
a
b
c
lyc
P3611
All bins
bins mapping EPRVs

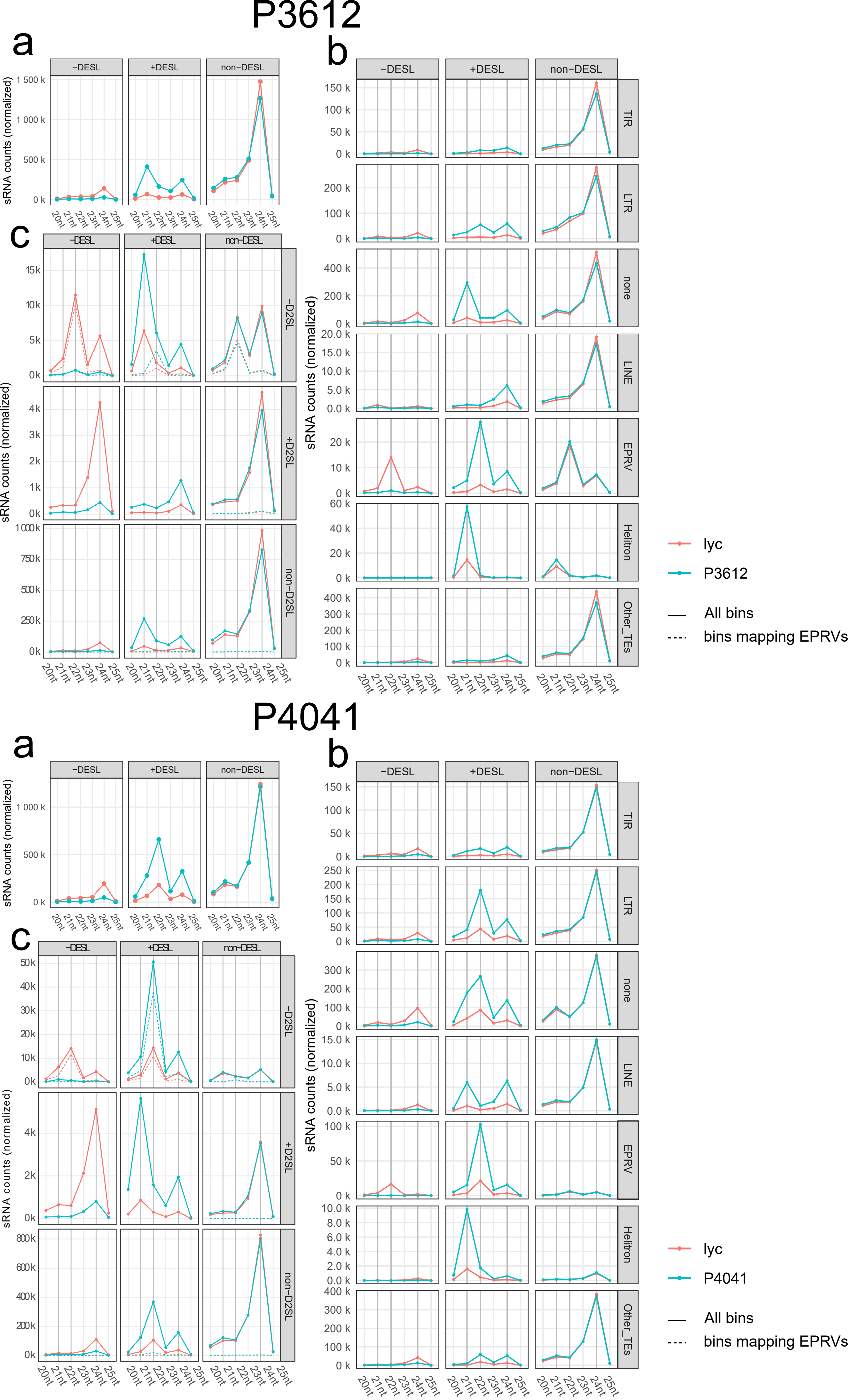
P3612
a
b
c
-DESL
+DESL
non-DESL
-D2SL
+D2SL
non-D2SL
TIR
LTR
none
LINE
EPRV
Helitron
Other_TEs
sRNA counts (normalized)
20nt
21nt
22nt
23nt
24nt
25nt
lyc
P3612
All bins
bins mapping EPRVs
P4041
lyc
P4041
All bins
bins mapping EPRVs

Figure S6

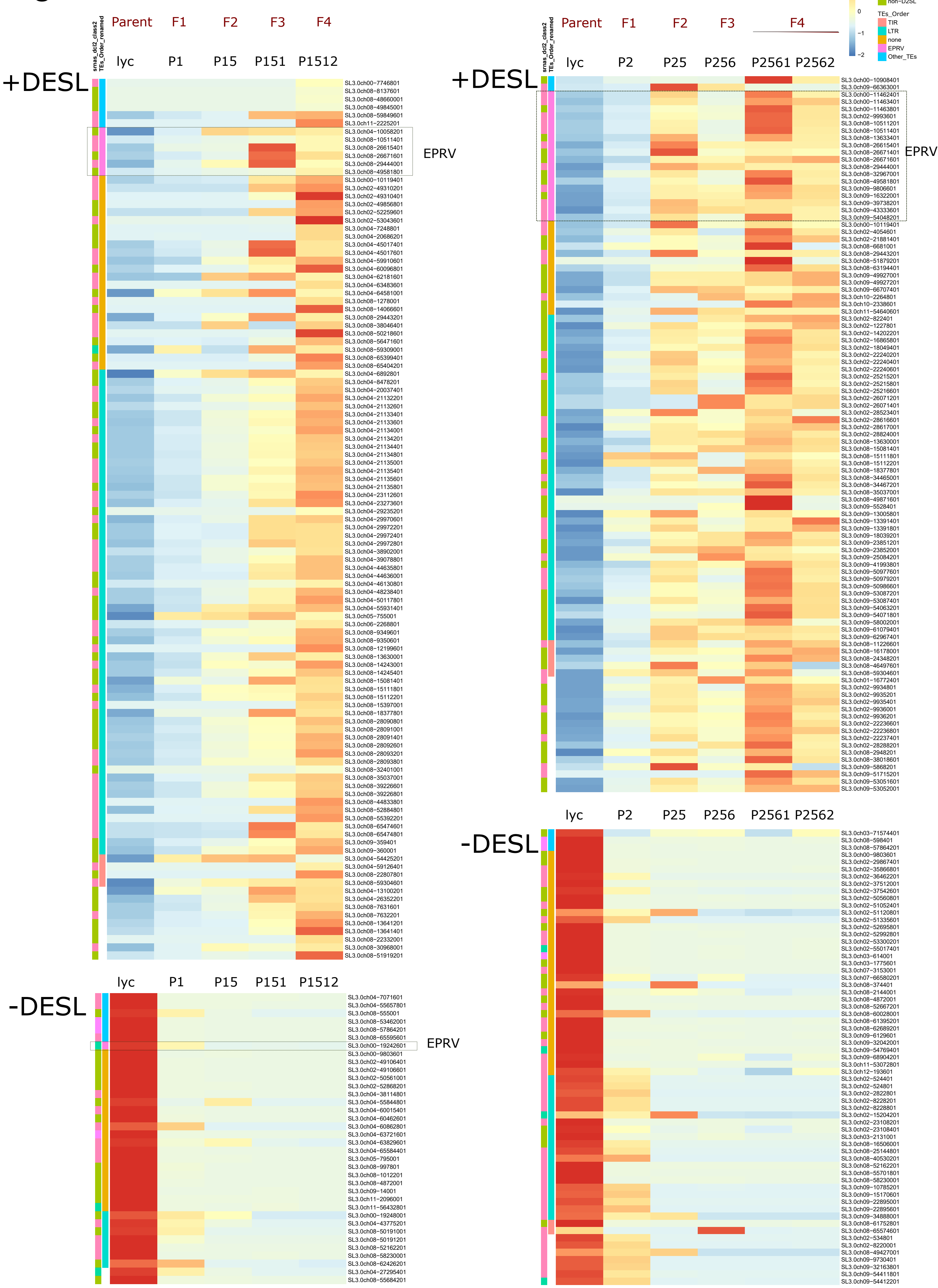
P1512
P2562
Parent
F1
F2
F3
F4
lyc
P1
P15
P151
P1512
P2
P25
P256
P2561
P2562
+DESL
-DESL
EPRV
srnas_dcl2_class2
TEs_Order_renamed
-D2SL
+D2SL
inbetween
non-D2SL
TEs_Order
TIR
LTR
none
EPRV
Other_TEs

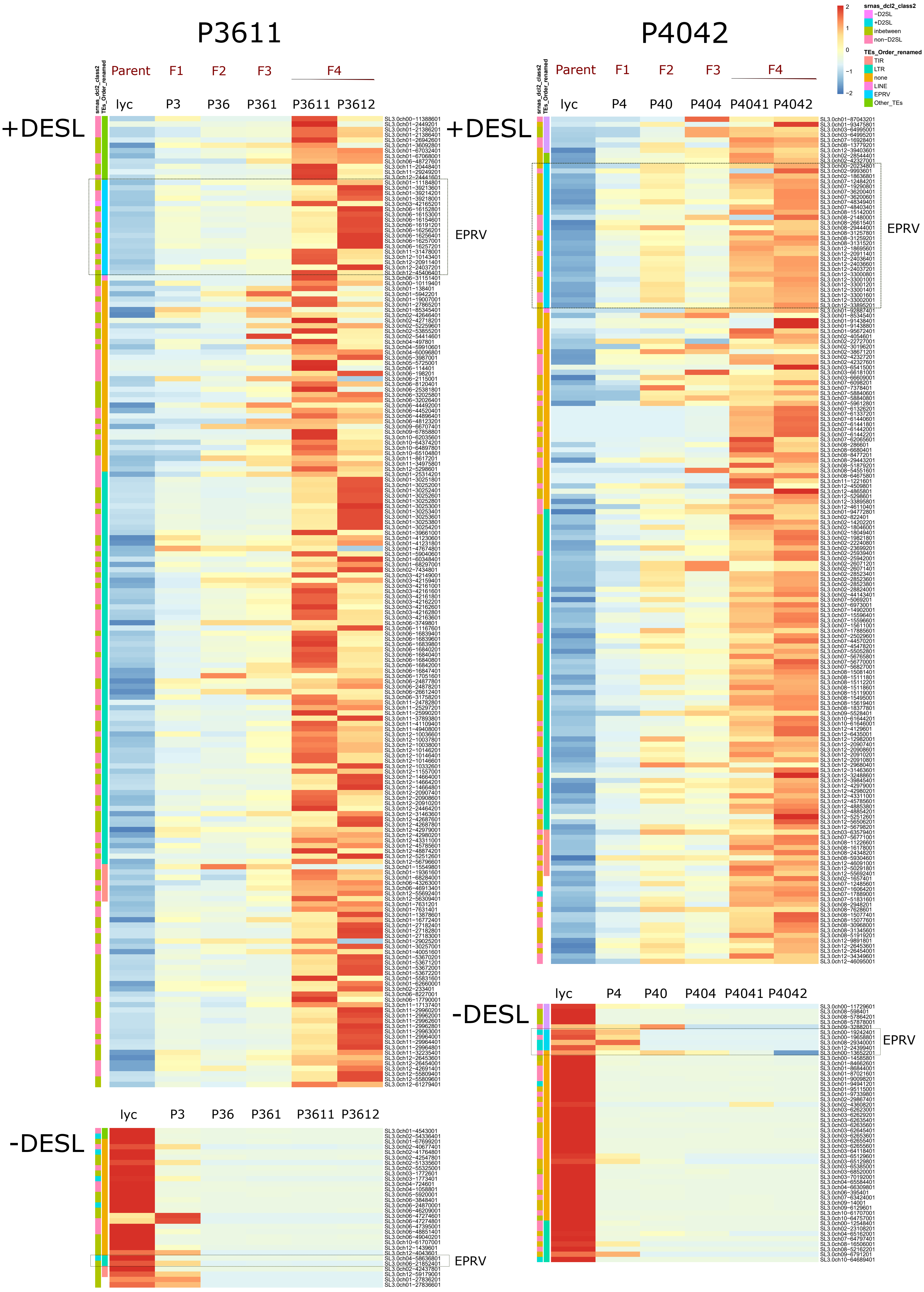
P3611
P4042
Parent
F1
F2
F3
F4
lyc
P3
P36
P361
P3611
P3612
P4
P40
P404
P4041
P4042
+DESL
-DESL
EPRV
srnas_dcl2_class2
TEs_Order_renamed
−D2SL
+D2SL
inbetween
non−D2SL
TIR
LTR
none
LINE
EPRV
Other_TEs
2
1
0
−1
−2

Figure S7

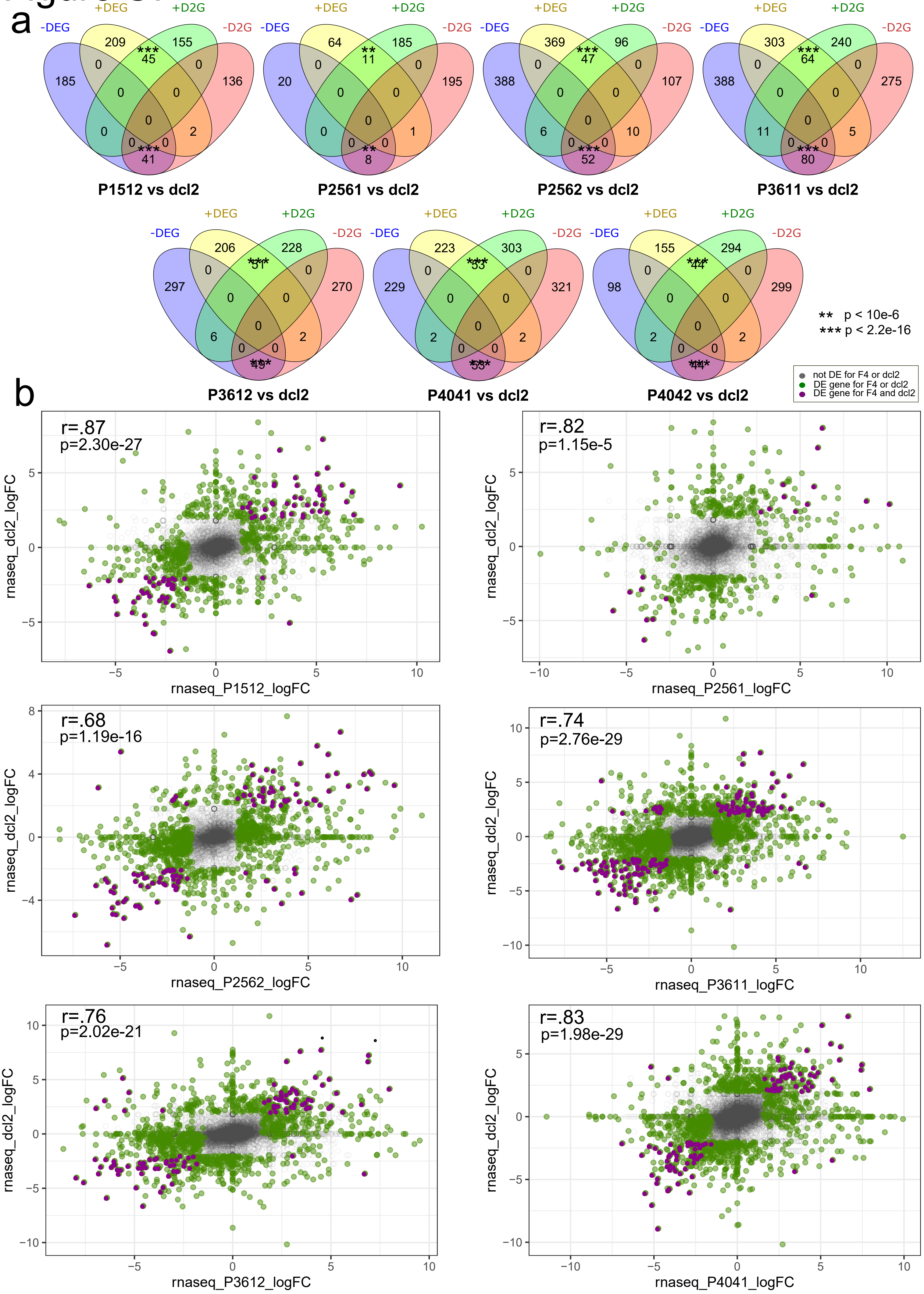
a
+DEG
+D2G
-DEG
-D2G
P1512 vs dcl2
P2561 vs dcl2
P2562 vs dcl2
P3611 vs dcl2
P3612 vs dcl2
P4041 vs dcl2
P4042 vs dcl2
** p < 10e-6
*** p < 2.2e-16
b
not DE for F4 or dcl2
DE gene for F4 or dcl2
DE gene for F4 and dcl2
r=.87
p=2.30e-27
r=.82
p=1.15e-5
r=.68
p=1.19e-16
r=.74
p=2.76e-29
r=.76
p=2.02e-21
r=.83
p=1.98e-29
rnaseq_dcl2_logFC
rnaseq_P1512_logFC
rnaseq_P2561_logFC
rnaseq_P2562_logFC
rnaseq_P3611_logFC
rnaseq_P3612_logFC
rnaseq_P4041_logFC
