# Supplementary material for: Interspecific hybridization in tomato influences endogenous viral sRNAs and alters gene expression
Source: Genome Biol. 2022 May 21;23:120. doi: 10.1186/s13059-022-02685-z (PMC9124383; doi:10.1186/s13059-022-02685-z)
Supplement: Supplementary file 4 — Additional file 4: Reviewhistory. [file 13059_2022_2685_MOESM4_ESM.docx]

Review History

**First round of review**

**Reviewer 1**

**Were you able to assess all statistics in the manuscript, including the appropriateness of statistical tests used?**

No, I do not feel adequately qualified to assess the statistics.

**Comments to author:**

This manuscript by Lopez-Gomollon and colleagues presents comparative analyses of gene expression and small RNA accumulation in 4th generation hybrids of Solanum lycopersicum and S. pennellii. The authors hypothesized that variations in transposon-derived small RNAs could be associated to transgressive phenotypes observed in the hybrid populations. However, and interestingly, they observe mostly a non-random distribution of differentially expressed genes and small RNA loci, thus indicating that hybridization triggers stable transgenerational genome-wide effects that are not necessarily linked to phenotypic variation. I find the manuscript very concise with a clear message, but perhaps the authors could make an additional effort to further elaborate on several key results.

Main concerns:

1. RNA sequencing allows a precise determination of parental contributions in hybrid siblings, and the analysis focused on homozygous regions. I understand the interest of using only homozygous regions for some of the analysis, but it was unfortunate to completely ignore the heterozygous regions. For example, it would be interesting to analyze potential effects of parental genome dominance, both in terms of gene expression and small RNA accumulation.

2. Small RNA analysis should include data from both parents, to investigate if the small RNA responses are essentially induced by hybridization, or already present in one of the parents. I only see data from the S.lycopersicum presented in the main figures and supplemental files.

3. As 22nt siRNAs are associated with post-transcriptional gene silencing, I was wondering if the authors also detect active transcription from EPRVs in the hybrids. This might allow mapping more precisely the elements contributing to siRNA biogenesis.

4. Previous work from the same group showed that miR6026 targets DCL2 in tomato, in a feedback loop mechanism that provides susceptibility to viral infections. It would be interesting to analyze the same pathway in this study and I am surprised it was completely ignored, as it could provide clues for how DCL2 activity is mis-regulated in a context of hybridization. Also, the abundance of DCL2-derived siRNAs triggered by miR6026 could be easily profiled using the datasets available.

5. The partial overlap with genes differentially expressed in dcl2 mutants is interesting but is not sufficient to conclude that DCL2 affects gene expression in the hybrids. The authors would need to introgess dcl2 mutant alleles or target mimics in hybrid populations in order to validate this hypothesis. Meanwhile, it would more reasonable to say that their data suggests that DCL2 is involved.

Additional comments:

- Not clear if the samples used for RNA-seq were the same used for small RNA-seq. This should be better clarified in the methods.

- Perhaps would be better if the y axis values in Figure 1d and 2a (and Additional figure 3a/b) are normalized to fraction of genome analyzed for each hybrid line, so that it does not give the wrong impression that some hybrids have more or less DEGs than others.

- The authors should test the statistical significance of the overlaps presented in all Venn diagrams.

**Reviewer 2**

**Were you able to assess all statistics in the manuscript, including the appropriateness of statistical tests used?**

Yes, and I have assessed the statistics in my report.

**Comments to author:**

In the manuscript, Lopez-Gomollon et al. reported their findings of sRNA changes in tomato hybrids. They found the sRNA from EPRVs and helitrons were differentially expressed, and the 22nt sRNA species were the mostly affected. These DCL2-dependent 22nt sRNAs were shown to regulate the expression of genes considerably in tomato hybrids. Overall, the manuscript was clearly written, and the results are interesting and informative. However, I feel the story is incomplete. It ends abruptly with many important questions not answered.

Major comments:

(1) The title of "Endogenous virus sRNA regulate gene expression in tomato hybrids" seems to be too general, without giving any specific information. I believe it (sRNAs regulate gene expression) is true without any experiments.

(2) The story ends abruptly. The finding that 22nt sRNA change correlates with gene expression in tomato hybrids is quite interesting. But this discovery is not enough to support a publication. They are many questions worthy of further investigation. What is the BIOLOGY? In other words, "23% of the genes upregulated in dcl2 (+D2Gs) also increasing in the F4s (+DEGs) and 27% of the downregulated genes in dcl2 (-D2Gs) decreasing in the F4s (-DEGs)" (lines 191-193), what are the function of these genes? Are there any very interesting genes? Do they contribute to important traits of tomato hybrids?

(3) All the analyses focused on the homozygous genomic regions, but here is no clear description on how the homozygous and heterozygous regions were identified, as there is not whole genome DNA sequencing for the F4 plants. If using RNA-seq data, how were the intergenic regions (major sRNAs generating regions) defined as homozygous or heterozygous?

Also, one related question is whether these identified DESLs and DEGs show any parental genome bias?

Minor comments:

(1) there are a few typos:

Line 134, Fig2.C to Fig. 2C.

Line 272, 65C to 65℃

(2) Some fonts in figures are hard to read, for instance, the figure 2d and e.

**Authors Response**

**Point-by-point responses to the reviewers’ comments:**

Reviewer #1: This manuscript by Lopez-Gomollon and colleagues presents comparative analyses of gene expression and small RNA accumulation in 4th generation hybrids of Solanum lycopersicum and S. pennellii. The authors hypothesized that variations in transposon-derived small RNAs could be associated to transgressive phenotypes observed in the hybrid populations. However, and interestingly, they observe mostly a non-random distribution of differentially expressed genes and small RNA loci, thus indicating that hybridization triggers stable transgenerational genome-wide effects that are not necessarily linked to phenotypic variation. I find the manuscript very concise with a clear message, but perhaps the authors could make an additional effort to further elaborate on several key results.

Main concerns:

1. RNA sequencing allows a precise determination of parental contributions in hybrid siblings, and the analysis focused on homozygous regions. I understand the interest of using only homozygous regions for some of the analysis, but it was unfortunate to completely ignore the heterozygous regions. For example, it would be interesting to analyze potential effects of parental genome dominance, both in terms of gene expression and small RNA accumulation.

*Reply: The main reason for our focus on homozygous lyc is technical: S. lycopersicum has a curated and well annotated genome and transcriptome, while this is not the case for S. pennellii. Our approach, therefore, was to use the well annotated lyc genome to look at details of the difference between the parents and the hybrid and to carry out an overview of the pen genome to see whether it was similarly affected by hybridisation. The relevant pen data are described in the text and in Additional Fig.3a-e. Any analysis of the F1 or heterozygous regions in the F4 would inevitably have been biased by the incomplete annotation of the pen genome. A further complication, the short length of sRNA reads, prevents unambiguous attribution to the parental contribution in heterozygous regions. By focussing on homozygous regions, we could attribute sRNA and RNAseq reads unambiguously to the respective parental genomes.*

*A second reason was because we wanted to differentiate our study from the many analyses of heterosis based on changes to gene expression and sRNA in the F1 (see paragraph starting on line 93). Our aim was to investigate changes to gene expression and sRNA that persist beyond the F1.*

2. Small RNA analysis should include data from both parents, to investigate if the small RNA responses are essentially induced by hybridization, or already present in one of the parents. I only see data from the S.lycopersicum presented in the main figures and supplemental files.

*Reply: We did include data from both lyc and pen in the original submission. The comparison of pen vs F4 for RNAseq and sRNAseq is in Additional Figure 3. We have now clarified the parent used for each comparison in the main text and in the title and legend of Additional Figure 3.*

3. As 22nt siRNAs are associated with post-transcriptional gene silencing, I was wondering if the authors also detect active transcription from EPRVs in the hybrids. This might allow mapping more precisely the elements contributing to siRNA biogenesis.

*Reply: In the RNAseq data we identified several EPRV, with 34 of them differentially expressed in at least one F4 plant but without statistically relevant association to DESLs. Below is a plot showing percentage (left) and number of TE (right) for differentially expressed EPRV and other TE groups. (***Please, see plots in the attached file "cover and responses to reviewers").*

*It is likely that the differentially expressed EPRV RNAs do not correlate with the DESL because they would be both a source and a target of RNA silencing. The primary RNA transcripts from the DESL would be degraded by DCL during sRNA biogenesis and would be targeted by the sRNA RISC during posttranscriptional RNA silencing. A likely consequence of these processes is that the EPRV DESL loci would not be expressed in the parents and that their RNAs would not accumulate in the F4 lines due to the posttranscriptional silencing.*

4. Previous work from the same group showed that miR6026 targets DCL2 in tomato, in a feedback loop mechanism that provides susceptibility to viral infections. It would be interesting to analyze the same pathway in this study and I am surprised it was completely ignored, as it could provide clues for how DCL2 activity is mis-regulated in a context of hybridization. Also, the abundance of DCL2-derived siRNAs triggered by miR6026 could be easily profiled using the datasets available.

*Reply: In summary: the levels of miR6026 are within the range of the two parents in the F4 progeny, the level of the miR6026-triggered sRNAs is reduced in some of the F4 lines (Additional table 2) and the level of DCL2 mRNA does not change consistently in the F4 lines (Additional table 1). These findings are not consistent with a model in which the phenotype of the F4 lines is due to a change in the level of DCL2. The evidence that miR6026 is unchanged relative to the parents is now included in Additional table 2 and the miRNA analysis is described in Material and Methods.*

5. The partial overlap with genes differentially expressed in dcl2 mutants is interesting but is not sufficient to conclude that DCL2 affects gene expression in the hybrids. The authors would need to introgess dcl2 mutant alleles or target mimics in hybrid populations in order to validate this hypothesis. Meanwhile, it would more reasonable to say that their data suggests that DCL2 is involved.

*Reply: We agree. Our conclusion is not that DCL2 is causal of the changes in gene expression, but that it is involved as implied in:*

*lines 189-191 “This analysis (Fig.4a,b, Additional file Fig.7a,b) revealed an extraordinary coincidence in the patterns of gene expression affected by dcl2 and the lyc x pen hybridisation both in terms of the gene identity and the quantitative effect on the expression level.”;*

*lines 197-199 “From these patterns we conclude that the hybridisation of lyc x pen perturbed the DCL2-mediated pathway of sRNA silencing and thereby influenced the profile of gene expression in the F4 progeny”.*

*In response to this comment, we also point out that the ‘partial’ overlap between the dcl2 and hybrid expressed genes is highly significant – see below. We are not saying that the only mechanism affecting gene expression in these hybrids involves DCL2 but this process does clearly account for a major component of the differentially expressed genes.*

Additional comments:

- Not clear if the samples used for RNA-seq were the same used for small RNA-seq. This should be better clarified in the methods.

*Reply: Samples for RNAseq and sRNA were independently isolated. This has been added in methods section.*

- Perhaps would be better if the y axis values in Figure 1d and 2a (and Additional figure 3a/b) are normalized to fraction of genome analyzed for each hybrid line, so that it does not give the wrong impression that some hybrids have more or less DEGs than others.

*Reply: To address this issue, we divided the number of + or - DEG by the total number of genes in the homozygous region for that specific plant. We have now updated Fig.1d, Fig.2a, Additional Fig.3a,b and legends accordingly.*

- The authors should test the statistical significance of the overlaps presented in all Venn diagrams.

*Reply: We have now included Fisher’s exact statistical test for the overlaps in the Venn diagrams, being the P-value <2.2e-16 for almost all cases, except P2561 that is p <10e-6. Fig.4 and Additional Fig.7 have been updated.*

Reviewer #2: In the manuscript, Lopez-Gomollon et al. reported their findings of sRNA changes in tomato hybrids. They found the sRNA from EPRVs and helitrons were differentially expressed, and the 22nt sRNA species were the mostly affected. These DCL2-dependent 22nt sRNAs were shown to regulate the expression of genes considerably in tomato hybrids. Overall, the manuscript was clearly written, and the results are interesting and informative. However, I feel the story is incomplete. It ends abruptly with many important questions not answered.

Major comments:

(1) The title of "Endogenous virus sRNA regulate gene expression in tomato hybrids" seems to be too general, without giving any specific information. I believe it (sRNAs regulate gene expression) is true without any experiments.

*Reply: we have changed the title to “Interspecies hybridisation in tomato influences endogenous viral sRNAs and alters gene expression”.*

(2) The story ends abruptly. The finding that 22nt sRNA change correlates with gene expression in tomato hybrids is quite interesting. But this discovery is not enough to support a publication. They are many questions worthy of further investigation. What is the BIOLOGY? In other words, "23% of the genes upregulated in dcl2 (+D2Gs) also increasing in the F4s (+DEGs) and 27% of the downregulated genes in dcl2 (-D2Gs) decreasing in the F4s (-DEGs)" (lines 191-193), what are the function of these genes? Are there any very interesting genes? Do they contribute to important traits of tomato hybrids?

*Reply: We have now added more information about the DEG in the text. 10% are transcriptional factors, including several genes involved in the phytohormone pathway, WRKY transcriptional factors, that are key regulators of biotic and abiotic stress or BBX/CONSTANS, that are central regulators of developmental processes, such as photomorphogenesis. We speculate that they could contribute to the transgressive phenotypes described in additional figure 1 but it is not a trivial undertaking to link genes, phenotypes and sRNAs.*

*The end of the paper coincides with a natural break in the analysis of these hybrid plants. The obvious next steps would be to identify the specific EPRV loci that are involved, the mechanisms associated with the differential expression of the sRNAs from these loci and whether the sRNAs have direct or indirect effects on gene expression. We have added some additional text to cover these points so that the ending does not seem so abrupt.*

(3) All the analyses focused on the homozygous genomic regions, but here is no clear description on how the homozygous and heterozygous regions were identified, as there is not whole genome DNA sequencing for the F4 plants. If using RNA-seq data, how were the intergenic regions (major sRNAs generating regions) defined as homozygous or heterozygous?

*Reply: We used the RNAseq data and applied a hidden Markov Model, as detailed in Material and Methods lines 316 to 326.*

*Even though RNAseq reads do not cover the whole genome, we have optimised the protocol to work out the genome map. As proof of concept, we applied this approach for the parental lyc and pen, obtaining a prediction of 99.6% lyc homozygosity for lycopersicum and 98.6% pen homozygosity for pennellii, as we showed in Figure 1b. We have now included a specific reference to the data from parental lines as a confirmation of the approach to the method section and in the main text.*

Also, one related question is whether these identified DESLs and DEGs show any parental genome bias?

*Reply: Our sample is small (n=7 F4 plants), but we do see a bias towards the pennellii genome representation, and also a higher degree of heterozygosity than would be expected in the F4 generation (lines 100 -103). Within the homozygous regions of lyc and pen, however, there were similar trends for DESL (lines 135- 139).*

Miner comments:

(1) there are a few typos:

Line 134, Fig2.C to Fig. 2C.

Line 272, 65C to 65℃

*Reply: fixed.*

(2) Some fonts in figures are hard to read, for instance, the figure 2d and e.

*Reply: fixed.*

**Second round of review**

**Reviewer 1**

The authors have addressed all my previous comments, and I have no further questions or concerns.

**Reviewer 2**

I am largely satisfactory with the revisions. I agree that "it is not a trivial undertaking to link, phenotypes and sRNAs", but to have a publication in Genome Biology is not trivial.

I am still quite confused on the assignment of homologous and heterozygous regions only using RNAseq data. In lines 341-343, "The assumption was that genotype patterns are relatively large (typically mega bases) containing multiple regions with transcriptional activity, i.e. the genotype of an interval between to transcriptional location of the same genotype is likely to be of the same genotype". I do not fully agree with this assumption as the majority of the genome are not transcribed. Is there any literature to support this? My suggestion is to name these regions as "homologous transcribed regions", which might be more accurate.

**Authors Response**

**Point-by-point responses to the reviewers’ comments:**

Reviewer #1:

The authors have addressed all my previous comments, and I have no further questions or concerns.

*Reply: Thank you.*

Reviewer #2:

I am still quite confused on the assignment of homologous and heterozygous regions only using RNAseq data. In lines 341-343, “The assumption was that genotype patterns are relatively large (typically mega bases) containing multiple regions with transcriptional activity, i.e. the genotype of an interval between to transcriptional location of the same genotype is likely to be of the same genotype”. I do not fully agree with this assumption as the majority of the genome are not transcribed. Is there any literature to support this? My suggestion is to name these regions as “homologous transcribed regions”, which might be more accurate.

*Reply: We agree with the reviewer that calling these regions “homozygous” or “heterozygous” may be not accurate. We would prefer not to use “homologous” as this may mislead the reader towards an evolutionary interpretation. Following reviewer suggestion, we have renamed the regions as “homozygous transcribed regions” or “heterozygous transcribed regions” in the manuscript.*

*The implication of the reviewer’s comment is that the accuracy of genotyping would be lower in the heterochromatin than the distal regions of the chromosomes because genes are less densely distributed in that region. In principle this caveat is correct. In practise, however, it is unlikely to have had a major effect because the lower gene density in heterochromatin is compensated by lower recombination frequency.*

*Based on the definition of chromatin regions using data from Wang, Nat Communications, 2020, we estimate that 14% of the tomato gene sequences are in the heterochromatin with the remaining 86% being in the relatively small genomic region (23%) corresponding to distal or euchromatin. We can be confident, therefore, that are transcribed region genotyping is sufficiently dense in the distal chromatin.*

*There is extensive literature to supporting the lower recombination rate in heterochromatin. It can be up to 1000-fold lower than for euchromatin, especially in interspecific tomato hybrids (Wang et al, Genetics, 2006; Tanksley et al, Genetics, 1992). Crossovers are originated from meiotic double stranded breaks (DSB) that are not repaired by homologous recombination. Meiotic DSBs are found to be common at gene promoters, which are AT-rich and nucleosome-depleted regions, mostly found in euchromatin (Choi et al., Genome Research, 2018). This reflects the final number of meiotic crossovers, that “mostly occur within gene-rich areas, close to gene promoter and terminator regions, where AT-rich DNA motifs are enriched and nucleosome occupancy is low” while “recombination cold spots are observed in heterochromatin rich centromeres and telomeres in most plant species “(from Wang et al, JXB, 2021).*

*RNAseq for genotyping has also been used for different purposes in other organisms (as example, Fan et al, Genome Research, 2018) and we extended that idea by using a hidden Markov model approach to identify the homozygous transcribed regions in the plants. Our assumption that the “genotype patterns are relatively large”, is based on the DSB interference, phenomenon first described in budding yeast that reduces the possibility of two DSB events occurring close to one another (Garcia et al., Nature, 2015). This reference has been now added to the method section.*

*We tested our hypothesis by applying this approach to both wild types, lyc and pen. As the number of genes in heterochromatin and euchromatin is similar for lycopersicum, pen and the F4 hybrids, we expect the same rate of uncertainty for parents and F4s. With our approach we identified for lycopersicum parent a 99.6% of the genome as homozygous transcribed regions, with a 0.3% as heterozygous and 0.1% as homozygous for pennellii. For the pennellii parent, we identified 98.7% of the genome as homozygous for pennellii, with a 0.7% heterozygous and 0.6% homozygous for lycopersicum (lower homozygosity for pennellii likely as consequence of pennellii genome being less polished than lycopersicum).*

*The results of our non-biased genotyping method for each parent gives us an estimation of the expected accuracy of this approach: 99.6% correct prediction for lycopersicum and 98.7% correct prediction for pennellii (Figure 1 b).*
